# Unraveling the Photoionization Dynamics of Indole in Aqueous and Ethanol Solutions

**DOI:** 10.1021/acs.jpcb.4c01223

**Published:** 2024-04-24

**Authors:** Gaurav Kumar, Michael Kellogg, Shivalee Dey, Thomas A. A. Oliver, Stephen E. Bradforth

**Affiliations:** † Department of Chemistry, University of Southern California, Los Angeles, California 90089, United States; ‡ School of Chemistry, Cantock’s Close, University of Bristol, Bristol BS8 1TS, U.K.

## Abstract

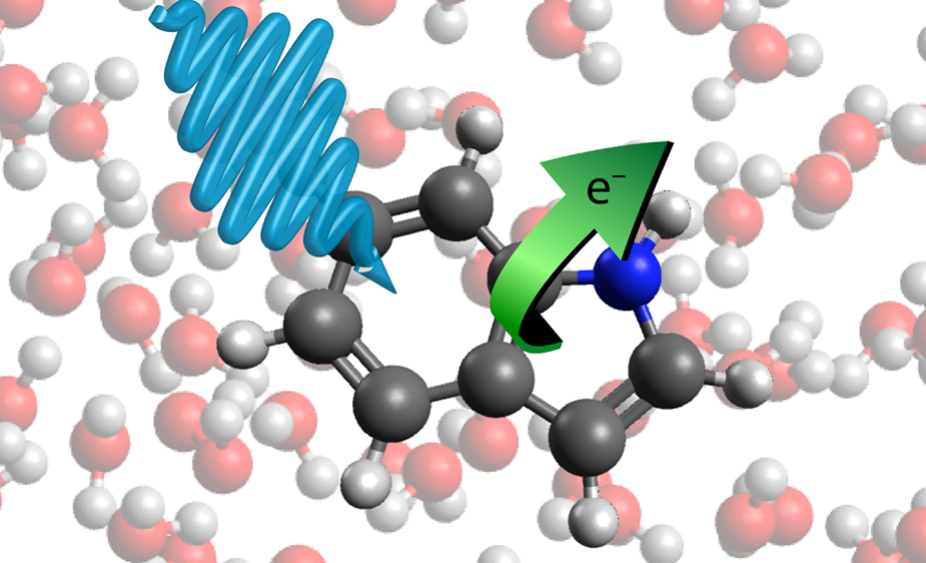

The photoionization dynamics of indole, the ultraviolet-B chromophore of tryptophan, were explored in water and ethanol using ultrafast transient absorption spectroscopy with 292, 268, and 200 nm excitation. By studying the femtosecond-to-nanosecond dynamics of indole in two different solvents, a new photophysical model has been generated that explains many previously unsolved facets of indole’s complex solution phase photochemistry. Photoionization is only an active pathway for indole in aqueous solution, leading to a reduction in the fluorescence quantum yield in water-rich environments, which is frequently used in biophysical experiments as a key signature of the protein-folded state. Photoionization of indole in aqueous solution was observed for all three pump wavelengths but via two different mechanisms. For 200 nm excitation, electrons are ballistically ejected directly into the bulk solvent. Conversely, 292 and 268 nm excitation populates an admixture of two ^1^ππ* states, which form a dynamic equilibrium with a tightly bound indole cation and electron–ion pair. The ion pair dissociates on a nanosecond time scale, generating separated solvated electrons and indole cations. The charged species serve as important precursors to triplet indole production and greatly enhance the overall intersystem crossing rate. Our proposed photophysical model for indole in aqueous solution is the most appropriate for describing photoinduced dynamics of tryptophan in polypeptide sequences; tryptophan in aqueous pH 7 solution is zwitterionic, unlike in peptides, and resultantly has a competitive excited state proton transfer pathway that quenches the tryptophan fluorescence.

## 1 Introduction

Indole is the ultraviolet-B light-absorbing chromophore of the important amino acid, tryptophan. The emission maxima and intensity (and thus fluorescence quantum yield) of tryptophan are used to study protein dynamics and associated structural changes^1,2^ since its emission is highly sensitive to the local environment.^1,3−5^ Indole is also a building block of eumelanin, a skin pigment, which is known for its photoprotective role in the ultraviolet part of the solar spectrum.^6,7^ Since the photochemical behavior of indole has been central to the understanding of important biological functions/pathways, it has been the subject of many molecular studies investigating its electronic structure and intricate excited state dynamics.^8−14^ There has been a recent resurgence of interest in using tryptophan fluorescence as a marker for more complex processes, for example, long-range energy transfer in microtubules.^15^ To underpin the growing prominence of these studies, it is essential that the underlying photophysics of its chromophore is firmly established.

The photophysics of isolated indole molecules is primarily governed by the low-lying ^1^ππ* (^1^L_a_ and ^1^L_b_ in Platt notation) and ^1^πσ* excited states, which in the gas phase fall within a 0.5–0.7 eV range.^8,12,16−19^ The ^1^L_a_ and ^1^L_b_ states possess significant oscillator strength, while the ^1^πσ* state is predicted to have a negligible absorption cross-section.^16,17^ The absorption band between 220 and 280 nm (Figure S2) is assigned to predominantly the ^1^L_a_ ← S_0_ transitions, while absorption to the ^1^L_b_ state contributes significantly at >280 nm. Population of the ^1^πσ* in the gas phase has two possible fates: repopulation of the ground state through a conical intersection (CI) at extended N–H bond lengths or N–H bond scission to generate indolyl radicals and H atoms.^13,19−21^

Flash laser photolysis studies in cyclohexane indicated an appreciable N–H bond dissociation yield at 265 nm,^22,23^ mimicking the gas phase dynamics at ≤263 nm; however, the complexity of the excited state processes increases when indole is dissolved in polar and more strongly interacting solvents such as water. Steady-state fluorescence studies have reported the energetic ordering of the ^1^L_a_ and ^1^L_b_ states are inverted in water and polar solvents such as ethanol compared to the gas phase.^9,16,24,25^ This originates from the greater permanent dipole moment of the ^1^L_a_ state, which is dynamically and more substantially stabilized by solvation than the ^1^L_b_ state, as detailed by ultrafast fluorescence up-conversion and TA studies by the groups of Chergui and Cerullo for tryptophan.^26,27^ Notably, these two studies concentrated on the fast interconversion between the low-lying ^1^ππ* states and did not investigate the photoionization channel.

Pioneering flash photolysis studies by Bent and Hayon demonstrated that the photochemistry of indole and tryptophan shared many similarities in aqueous solution^28^ and additionally demonstrated that a variety of different photochemical pathways were active, including photoionization. This important study stimulated many subsequent investigations to understand the mechanism of ionization,^29−34^ including those by Lee and Robinson who demonstrated that the photoionization process was one-photon driven,^35^ as opposed to multiphoton ionization.

The long-time solvated electron quantum yield (Φ_e–_) of indole in water was studied by several different experimental methods. Bernas et al. and Katoh estimated similar photoionization thresholds of 285 and 290 nm, respectively.^36,37^ Katoh demonstrated that the Φ_e–_ remained constant between 250 and 290 nm at 0.2 and Saito et al. reported Φ_e–_ = 0.27 at 266 nm.^23^ At wavelengths shorter than 250 nm, Katoh reported that the Φ_e–_ increased and reached 0.26 at 220 nm. Studies by Zechner et al. indicate the Φ_e–_ channel continues to become even more prevalent at 214 nm (0.32), with McGimpsey et al. reporting a yield of ∼0.3–0.4 at 193 nm.^38^

Peon et al. studied the photoionization dynamics of aqueous indole using ultrafast transient absorption (TA) spectroscopy with 260 and 262 nm excitation.^14,39^ They reported the formation of solvated electrons within their instrument response of 200 fs, which is considerably faster ionization and subsequent solvation compared to that observed in the UV photoionization of other heteroaromatic molecules in water.^40,41^ They suggested that the immediate generation of solvated electrons could arise from the direct transfer of the photoelectron to a trapping site in water without the formation of an intermediate delocalized electron in the conduction band, a view also shared by Kevan and Steen.^39,42^ A subsequent study by Bizjak et al. using 270 nm excitation with 80 fs time resolution reported prompt photoionization, followed by more typical ∼1 ps solvation dynamics for a solvated electron, and estimated a 45 ± 5% quantum yield for the channel.^25^ Ultrafast studies of indole at air/water interfaces indicated that photoionization was an active pathway and promptly generated partially solvated electrons at the water surface.^43^

Sobolewski and Domcke performed ab initio calculations on indole–(water)_*n*_ clusters (1 ≥ *n* ≥ 3) to explore the influence of microsolvation.^17^ Their calculations predicted the ^1^πσ* state evolves into a hydrated electron hydrogen bonded to the indole cation^17^ and extrapolated an adiabatic excitation energy of 4.35 eV, in good accord with experimentally determined values.^36,37^ Despite the theoretical prediction in water clusters, experiments on small gas phase indole–(water)_1–2_ clusters did not reveal any signatures of N–H bond fission dynamics or indole photoionization.^37,44−46^

The reported triplet quantum yield reported for indole in water is surprisingly high (0.23–0.35),^46,47^ with the mechanism of its formation and lifetime (microsecond and millisecond) remaining contentious.^48−51^ This mechanism has been attributed to “typical” intersystem crossing (ISC), recombination of indole cations with solvated electron photoproducts,^49,50^ or impurities in solutions.^51^ In contrast, gas phase time-resolved photoelectron experiments show no evidence for triplet indole formation within 1.2 ns.^13,20^ The lack of ISC in the gas phase is re-enforced by *ab initio* calculations by Serrano-Andrés and Roos,^24^ who revealed that there are no ^3^*n*π* states in indole within the near-UV region, and thus ISC following El-Sayed’s rules from the optically prepared ^1^ππ* states is unlikely. The associated energy levels for the relevant excited states in the gas phase are given in Figure S1.

In earlier work, we applied the emerging technique of time-resolved liquid jet photoelectron spectroscopy (LJ-PES) to the excited state dynamics of indole at 266 and 292 nm. One surprise from our study was the fact that all LJ-PES features, including those assigned to the ^1^L_a_ state, were observed to be kinetically impacted by known solvated electron quenchers.^52^ Here, we investigate the influence of solvation on the photophysics of indole using ultrafast TA spectroscopy for multiple pump wavelengths with 70 fs time resolution at 266/268 and 292 nm and 240 fs at 200 nm. We utilize a broadband near-UV to visible white light supercontinuum probe, which offers greater insights into the indole dynamics compared to earlier TA experiments studied which utilized two similar (266/262 nm) pump wavelengths and single wavelength probe detection^14,39^ or a single pump wavelength study (270 nm) using a narrower wavelength probe region.^25^ These data are also complemented by ab initio calculations to support spectral assignments. Part of our motivation was to understand the pump wavelength dependence of the ionization channel and whether this facet of the photophysics is responsible for the diminished fluorescence quantum yield when the indole chromophore is water-exposed in peptide sequences or proteins.^1,2,53,54^ Through a comprehensive study, we propose a new photophysical picture of this important UV chromophore, bringing together previous disparate findings from decades of study, and demonstrate that the photoexcited ^1^ππ* state forms a dynamic equilibrium with photoionized indole cation–electron ion-pair products. The ion-pair dissociates into free charges on a nanosecond time scale draining the equilibrium with the ^1^L_a_ state, and the rate of this nonradiative process dictates the ^1^L_a_ fluorescence lifetime. Conversely, 200 nm excitation photoprepares the ^1^B_a_ (or higher energy states) that ballistically ejects an electron into the bulk solution away from indole. Remarkably, photoionization is only a competitive excited state pathway in aqueous solution but not in polar ethanol solution.

## 2 Results

### 2.1 Photophysics of Indole in Ethanol Solution

**Figure 1:**
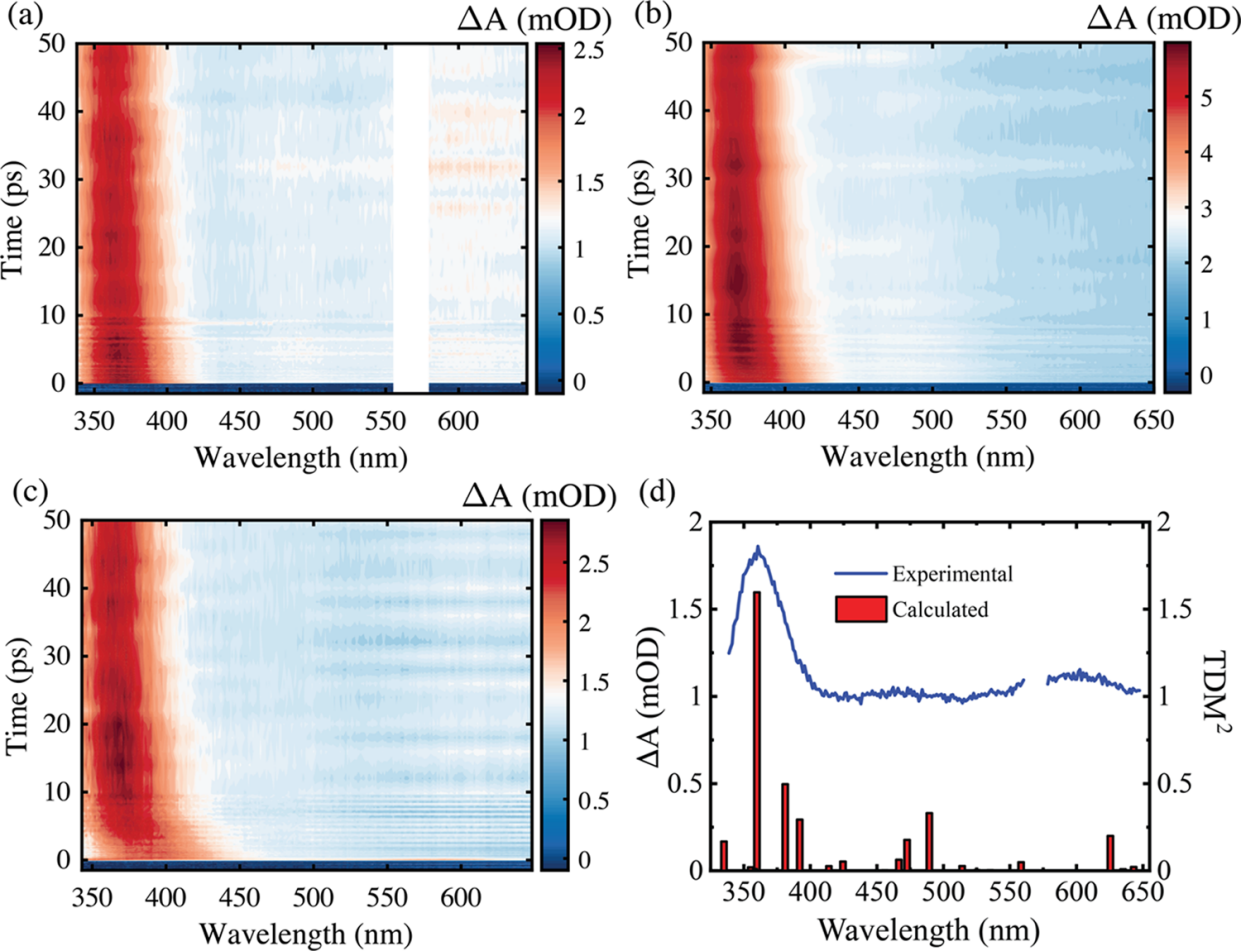
False color contour transient absorption spectra of indole in ethanol measured with excitation wavelengths for (a) 292 nm, (b) 266 nm, and (c) 200 nm excitation. Note that on this color scale, blue corresponds to 0 mOD signal. (d) Comparison of TA spectrum for 292 nm excitation averaged between 450 and 500 ps (blue line) and computed (EOM-CCSD/aug-cc-pVDZ) S_n_ ← ^1^L_a_ ESA transitions (red sticks). The probe region between 560 and 580 nm was removed from the experimental spectrum in panels (a) and (d) due to scatter from the harmonic of the pump beam.

**Figure 2:**
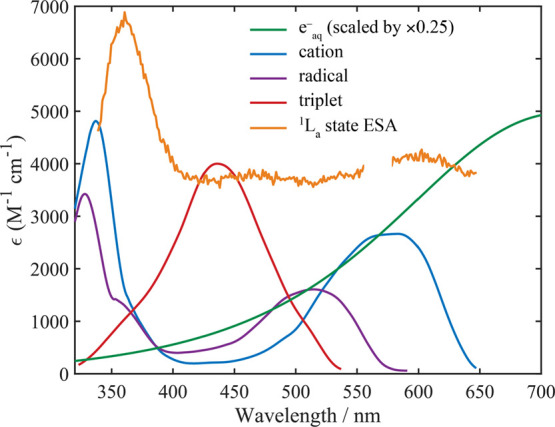
Experimentally measured absorption spectra of indole cation,^29^ radical,^29^ triplet,^28^ solvated electron species in water,^57^ and ^1^L_a_ state ESA in ethanol (this work). The solvated electron spectrum is scaled by 1/4, and the extinction coefficient has been corrected using the recent re-evaluated value.^58^ The triplet extinction coefficient was obtained from ref. (22), and the ^1^L_a_ ESA spectrum extracted from 450 to 500 ps TA data was scaled using the extinction coefficient from ref. (14). Note that there are expected to be differences in the ^1^L_a_ ESA spectra in water and ethanol.

The experimental and theoretical methods employed in this work and details of materials used are provided in the Supporting Information. Figure 1a–c displays two-dimensional false color contour maps of the transient absorption spectra of indole dissolved in ethanol for three different excitation wavelengths. The reported adiabatic ionization threshold of indole in ethanol is 4.85 eV (256 nm),^36^ indicating that indole will not be ionized with 267 or 292 nm excitation, but is expected to do so at 200 nm (6.2 eV). In the 200 nm pump data, there is an intense feature in the range of 350–450 nm with a maximum of ∼360 nm, which narrows and shifts toward shorter wavelengths with increasing pump–probe time delay. There is also a weaker broad feature present throughout the probe window that does not exhibit any time evolution within the 750 ps time delay window of our experiments. If the anticipated prompt ionization channel were active, this region should be dominated by the well-known strong absorption of the solvated electron signal peaking at ∼680 nm.^55^ The lack of an obvious solvated electron signal or indole cation (reference spectra are displayed in Figure 2) is surprising and indicates that despite being energetically feasible, indole does not seem to ionize to any significant extent upon 200 nm irradiation in ethanol, despite prior evidence from reaction with N_2_O which is a known solvated electron scavenger.^36^ Similar conclusions are also drawn from 266 and 292 nm pump TA data (Figure 1b,c) and time-correlated single photon counting (TCSPC) measurements (see Section 2.3). The TA data can instead be assigned to parent indole excited state absorption (ESA) using our calculated S_n_ ← ^l^L_a_ indole ESA spectrum (see details for calculation methodology in the Supporting Information). The agreement with 292 nm TA data is good (overlaid in Figure 1d) and spectrally identical to the 200 and 266 nm experimental data at *t* > 30 ps.

The main dynamical observation from these spectra is the excitation wavelength-dependent shifting of the parent ESA at wavelengths shorter than 450 nm, which at *t* > 30 ps maximizes at ∼360 nm. This spectral shift is largest for 200 nm (30 nm shift) followed by 266 nm (10 nm shift) and 292 nm (8 nm shift). In our TA studies of indole in water (vide infra) and prior studies of tryptophan by Cerullo and co-workers,^26^ the wavelength shift of the 375–340 nm feature is a key signature of the population transfer between the ^1^L_b_ and ^1^L_a_ electronic states and *independent* of the pump excitation wavelength. In ethanol, the time scale and magnitude of the wavelength shift for this band is proportional to the pump energy used (evident in spectral slices—Figure S4), and so evidently there must also be some degree of vibrational cooling also superimposed on the observed spectral dynamics. After these dynamics are complete, the TA spectra uniformly decay at all probe wavelengths matching the known indole fluorescence lifetime in ethanol determined by Gryczynski et al.,^56^ and illustrated by kinetic slices in Figure S4.

### 2.2 200 nm Photophysics of Indole in Water

In water, the ionization threshold for indole with respect to solvated electron production has been reported over the range 4.28–4.35 eV (290–287 nm),^36,37^ which suggests that photoionization should be an active reaction channel with 268 and 200 nm excitation but perhaps not for 292 nm (4.24 eV). More recent determinations from liquid jet photoelectron spectroscopy are consistent: we have reported vertical and adiabatic ionization energies, with respect to vacuum, for tryptophan as 7.3 and 5.9 eV.^59^ The value of vertical ionization energy is within error for aqueous indole as determined by resonant two-photon ionization^52^ and an X-ray PES study.^60^ Therefore, 200 nm (6.20 eV) excitation exceeds the thermodynamic threshold for generating solvated electrons of indole in water.^37^

**Figure 3:**
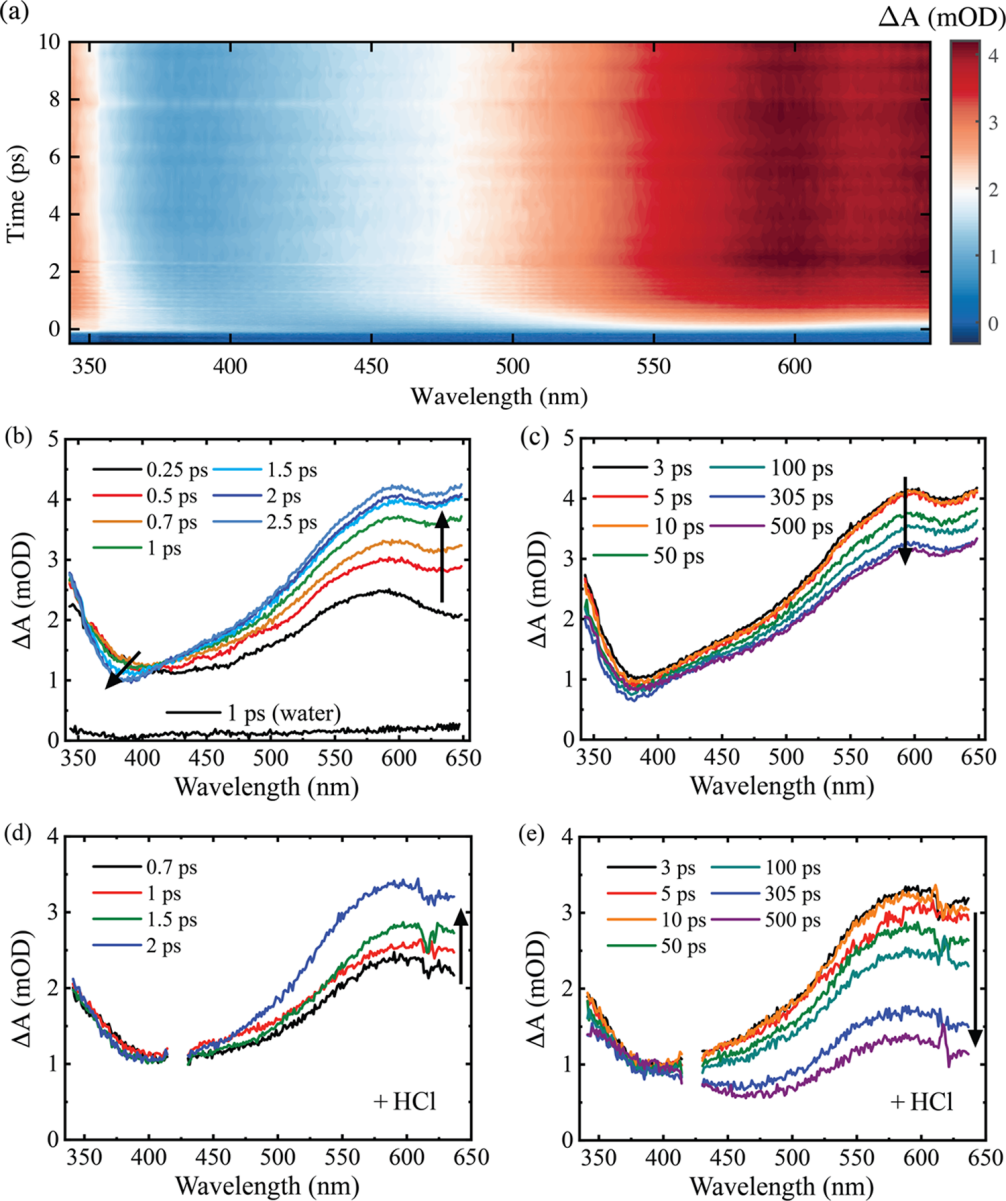
(a) False color contour plot of the 2D transient absorption data for 16 mM indole in water using 200 nm excitation between 0 ≤ *t* ≤ 10 ps; spectral slices at a series of (b) early and (c) later time delays. Spectral slices for the aqueous indole (10 mM) for 200 nm excitation when 0.2 M HCl is added for (d) early and (e) longer time delays. The signal in the 415–430 nm region in panels (d) and (e) were removed due to pump scatter. The arrows illustrate the main observed spectral rises, decays, and shifts.

Figure 3 displays the pump-induced transient absorption spectra of indole in water for 200 nm excitation. The contour plot in Figure 3a depicts the transient absorption data set up to 10 ps. Figure 3b,c displays TA spectra for specific pump–probe time delays and the evolution of transient species. We choose to break this into two distinct time-delay epochs: first, when the transient absorption at λ > 500 nm rises, and second, when the overall signal decays. Three key features stand out: (i) The most intense band is in the region of 500–650 nm and the signal at 650 nm rises more in 1 ps than the peak initially seen near 575 nm (Figure 3a); (ii) the region <375 nm appears immediately and reveals only the red-most edge of an ESA band which appears to peak at shorter wavelengths than observed in ethanol. The long wavelength edge of this band shifts/narrows by ∼10 nm over a few picoseconds (as seen by a shift in the absorbance minimum from 394 to 383 nm); (iii) after 1 ps, a small but notable shoulder appears in the range of 400–475 nm (maximum ∼440 nm); this shoulder is increasingly obvious at longer time delays (Figure 3c). After 2 ps, other than the ∼440 nm shoulder, the remainder of the spectrum shows a slow and partial decay stabilizing by 500 ps (Figure 3c).

The several picoseconds rise at 650 nm in the red part of the TA spectra is consistent with the formation of solvated electrons; for fully solvated electrons, i.e., spatially separated from the parent molecule, the signal should peak at 720 nm (outside the probe window) and exhibit a near-IR to visible blue-shift upon solvation in water on a hundreds-of-femtoseconds time scale. In contrast to the situations we will encounter in Section 2.3, 200 nm excitation of indole in water leads to clear-cut photoionization–generation of indole cations and entirely separated solvated electrons. To confirm the presence of solvated electrons, H^+^ electron scavenging experiments were conducted using 0.2 M HCl. Under these conditions, indole remains in the neutral state, and 0.2 M does not lead to acid-catalyzed polymerization of ground-state indole.^61^ In acid solution, as shown in Figure 3e, a large part of the red portion of the spectrum (kinetics probed at 633 nm shown in Figure S6) is quenched, confirming the presence of solvated electrons. The kinetics also reinforce this assignment and return a quenching rate constant *k*_quench_ = 1 × 10^10^ M^–1^ s^–1^, consistent with the known bimolecular scavenging rate of 1.3 × 10^10^ M^–1^ s^–1^ at 0.2 M H^+^ concentration.^62,63^ However, significant signal intensity remains across the rest of the spectrum with prominent bands at >340 and 575 nm. There are also weak transient features that appear at 367 and 400 nm which rise at *t* > 300 ps. Their assignment is discussed in the context of 268 nm proton quenching data.

Each ejected electron will be accompanied by its geminate partner, an indole cation, which has a weaker absorption cross-section than e^–^_aq_ (see Figure 2). The cation is expected to have a broad peak at 580 nm (as evident in the earliest time slice of data in Figure 3a) and an intense absorption at wavelengths shorter than 375 nm (Figure 2).^28,29^ When examining the indole data in acidic solution at *t* > 500 ps (Figure 3d), the cation signature is clearest and compares favorably with the indole cation spectrum reported in pulsed radiolysis experiments.^29^ To further reinforce the cation signature assignment, we calculated the electronic absorption transitions for both the indole cation radicals. The latter is a possible product formed by the deprotonation of the indole cation or direct photodissociation of the parent indole molecule. The calculated CASPT2/aug-cc-pVDZ absorption spectra for both species are displayed in Figure S7, and we find good agreement with the bands observed in pulsed radiolysis spectra (shown in Figure 2) and best agreement in our experiment with the indole cation after most solvated electrons have been quenched (Figure 3e).

There is a modest amount of blue-shifting in the TA spectrum for wavelengths shorter than 375 nm (*t* < 2 ps in Figure 3a,b), which is a signature of parent indole ESA and the aforementioned ^1^L_b_ → ^1^L_a_ population transfer.^26^ It signifies that some portion of the excited state indole molecules do not immediately photoionize. We attempted to reconstruct the main features in the experimental spectrum acquired at 200 nm by taking the sum of the indole cation^29^ and the solvated electron to recreate the 500 ps experimental TA spectrum (Figure S8). While the agreement is reasonable, there is significant intensity missing in the range of 400–550 nm in the constructed spectrum, and this can partially be explained by the presence of some parent ESA (displayed in Figure 2 for indole in ethanol). The indolyl radical was discounted from inclusion as deprotonation of the indole cation is known to occur on a microsecond time scale.^14,64^

### 2.3 268 and 292 nm Photophysics of Indole in Water

**Figure 4:**
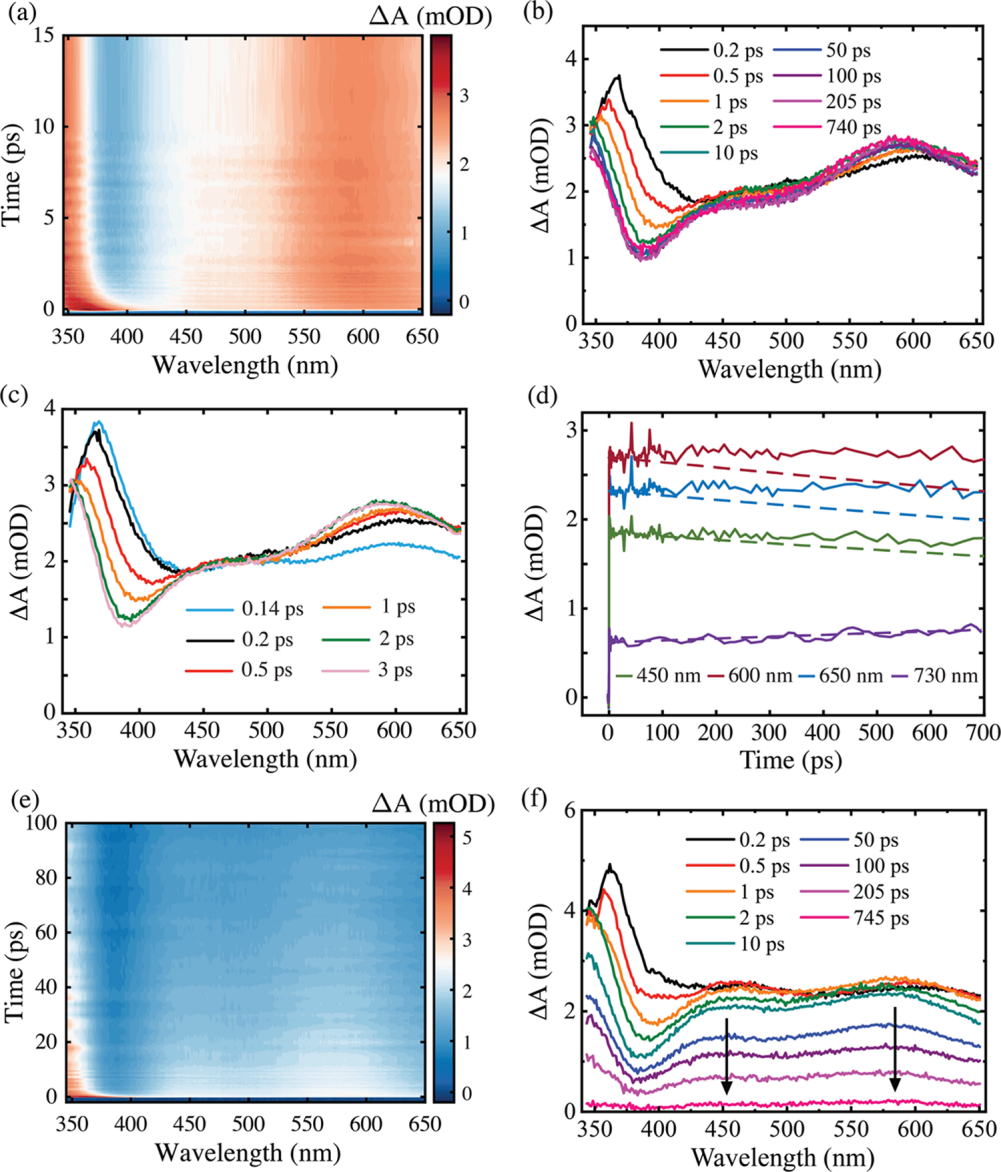
(a) False color contour plot of the full 2D transient absorption data set of 17 mM indole in water at 268 nm. Spectral slices at a series of time delays for (b) entire time delay range and (c) early time delays. (d) Kinetics associated with several key probe wavelengths where 730 nm data acquired separately as shown in Figure S15. (e) Contour plot of the TA data set when 0.5 M KNO_3_ was added to the 10 mM aqueous indole and (f) spectral slices for aqueous indole with 0.5 M KNO_3_. Dashed lines in panel (d) correspond to the 4.56 ns fluorescence lifetime of the ^1^L_a_ state of indole in water determined via TCSPC measurements. Note the different time axes in panels (a) and (e).

We now turn our attention to examining data on exciting indole at 268 nm in aqueous solution. This wavelength excites the ^1^L_a_ and ^1^L_b_ electronic states of aqueous indole in an approximately 3:1 ratio based on their relative absorption cross sections.^65^Figure 4a displays a false color contour plot showing our transient absorption spectrum for 268 nm excitation of indole in aqueous solution. Spectral slices for representative time delays are shown in Figure 4b. Our key observations from the spectra are (i) there are three distinct regions based on the signal intensity. The blue region of the spectrum is the most intense, followed by the red portion and then the region between 410 and 510 nm which shows a plateau at intermediate absorbance. (ii) The maximum of the electronic band in the blue edge shifts toward a shorter wavelength by 20 nm and is complete by a pump–probe delay of ∼2 ps, a far more marked wavelength shift than observed at a 200 nm pump. (iii) The red part of the spectrum peaks at 580 nm after a ∼20 nm spectral blue-shift upon a similar time scale, as evident in early time delay TA slices (Figure 4c) and kinetics at 600 and 650 nm (Figure S14). These dynamical shifts in the red part of the TA spectra are not evident in ethanol data (Figure S4), which is a solvent for which we have demonstrated indole photoionization does not occur, and ^1^L_b_–^1^L_a_ population transfer does not have associated spectral signatures in this TA wavelength region.

The TA data of indole in water for 268 nm excitation are different from 200 nm in many aspects. The shifting at the blue edge of 268 nm pump data is far more prominent than at 200 nm and reflects that the TA signal is dominated by the parent ESA. The shift and time scales are very similar to the data reported by Cerullo’s group for tryptophan in water, and again we assign it to ^1^L_b_ → ^1^L_a_ population transfer.^26^ The bands centered at 450 and 580 nm evident after *t* > 1 ps are dominated by ^1^L_a_ state ESA, based on the high-level calculations including water solvent by Garavelli and co-workers.^26^ Another striking difference is in the dynamics associated with the red part of the spectrum, which exhibits a small but significant rise and blue shift on a ∼ps time scale (as shown in Figure 4c), which must originate from the formation of a new, presumably dipolar, species that undergoes dynamic solvation, but not from separated electron formation (based on the kinetics shown at 600 and 650 nm, see Figure S14a). Given the lack of evidence for immediate ejection of an electron into bulk water, our proceeding results and analysis, and precedent in the literature for other systems,^63,66^ we propose that this species corresponds to a contact ion-pair of Indole^+^:e^–^ ([Ind^+^:e^–^]). Contact pairs involving electrons also have been invoked in the ionization of other aqueous organic molecules^67^ and have analogs to charge-transfer states in the photochemistry of aqueous inorganic anions.^39,68^ Electrons in such pairs with their parent molecule exhibit more modest spectral shifting^67^ than solvated electrons ejected further into solution.

The longer-term kinetics associated with 650 and 600 nm are displayed in Figure 4d and remain fairly static across the delay range studied, in accord with prior studies by Peon et al. for similar pump and probe wavelengths.^14,39^ However, the kinetics at 450 nm exhibit a slow decay and match more closely the ^1^L_a_ state fluorescence decay as determined by TCSPC (overlaid dashed line), only deviating significantly at *t* > 200 ps. TA data acquired in the near-IR region are shown in Figure S15. These data show that at 730 nm, near the peak of the solvated electron absorption band, the dynamics differ significantly (Figure 4d) and notably exhibit a slow *rise* that matches the ^1^L_a_ state fluorescence lifetime. The nanosecond rise in kinetics at this important probe wavelength *finally* indicates separated solvated electrons are formed and is the first strong piece of evidence in our data supporting indole photoionization (to separated charges) at 268 nm. To summarize this key result, from the range of kinetics observed at different probe wavelengths, it is evident that along with the ^1^L_a_ excited state of indole, other species such as the solvated electron must be generated and have different dynamics to rationalize the complex temporal evolution of different wavelengths in the TA data. This contrasts with data acquired in ethanol, where the entire TA spectrum is attributed to indole ESA and uniformly decays by 15% over the first 750 ps (see Figure S5). The rich wavelength-dependent dynamics of indole in water upon 268 nm excitation was not observed in prior TA studies by the groups of Kohler and Riedle, as only a narrower set of probe wavelength ranges were examined and/or limited maximum time delay.^14,25,39^

To search for further evidence that electrons are generated upon 268 nm excitation of indole, we performed electron scavenging experiments using a nitrate salt, KNO_3_, and a strong acid, HCl. Both NO_3_^–^ and H^+^ are known diffusively to quench separated solvated electrons but may also scavenge electrons inside contact or caged pairs.^69,70^Figure 4e displays the transient absorption data of aqueous indole with 0.5 M KNO_3_ as a false color contour plot, the same data are replotted as a set of time slices in Figure 4f, and control data at the same ionic strength using 0.5 M KCl are found in Figure S14c,d. If separated solvated electrons were produced, we would expect to see a significant differential change to the transient absorption spectrum at λ > 500 nm (as we did for 200 nm excitation in water) upon their removal.^14,37^ Counter to our expectations, the intensity of the spectra is dramatically quenched across all probe wavelengths starting at the earliest time delays, and by 745 ps, the signal has almost entirely decayed to baseline. We recognize that quenching at early times requires NO_3_^–^ to diffusively quench something other than separated solvated electrons at 268 nm, likely the aforementioned contact ion pair. As a result, TCSPC experiments using 260 nm excitation with KNO_3_ were used to determine if the indole fluorescence is also sensitive to nitrate (see Figure S10). These experiments surprisingly revealed very efficient dynamic quenching of the indole ^1^L_a_ state fluorescence, but interestingly, with a rate constant matching 7.6 × 10^9^ M^–1^ s^–1^, the known NO_3_^–^ scavenging rate with solvated electrons at infinite dilution.^71^ TCSPC experiments of indole in ethanol, a solvent that does not support indole photoionization, however, showed no change in the fluorescence lifetime upon the addition of NO_3_^–^ (Figure S13), demonstrating that nitrate does not *directly* interact with the ^1^L_a_ state of indole. We return to the mechanism for the complete quenching by nitrate of all species present in the TA spectra in Section 3.

**Figure 5:**
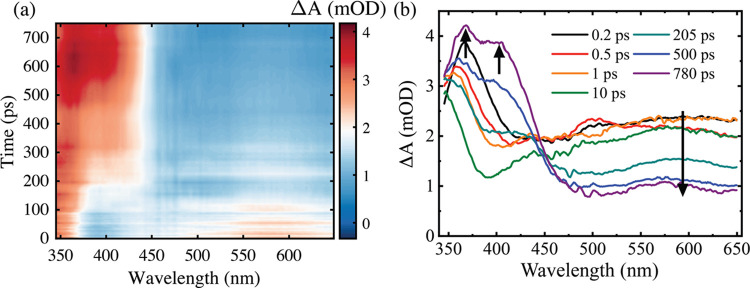
(a) Contour plot of the full 2D transient absorption data set of 10 mM aqueous indole when 0.2 M HCl is added to the solution at 268 nm excitation. (b) Spectral slices at a series of time delays.

The addition of 0.2 M HCl to indole solutions produced entirely different TA spectra, as illustrated in Figure 5. The various features present in the TA spectra are comprised of an admixture of several different species. The notable effect of the spectrum on the addition of protons to the solution (Figure 5a) is the intensity at λ > 475 nm is dramatically reduced, again indicative that electrons are available for scavenging. On a similar time scale to the decrease in the initially induced ESAs, two new features rise after ∼200 ps at 367 and 400 nm (marked with arrows in spectral slices displayed in Figure 5b). At longer pump–probe delays (e.g., 780 ps), a band peaking at 580 nm is clearly visible (Figure 5b), associated with the indole cation, as observed for 200 nm excitation (Figure 3d).

We next consider the origin of the new electronic bands centered at 367 and 400 nm in acidic indole solution (Figure 5b). Proton scavenging of electrons forms a neutral H atom, with the equilibrium for the reaction greatly favoring the hydrogen atom product.^72^ After 10 ps, the 400 nm band rise matches the decay at the 610 nm band (see Figure S17). Therefore, it is possible that once the protons have scavenged electrons from the contact pair, the resulting hydrogen atom can subsequently react with indole cation to form an adduct, as observed for the reaction between nascent photoproducts in our study of *para*-methylthiophenol.^73^ Bent and Hayon experimentally observed a similar transient in 265 nm flash photolysis studies of acidic indole (pH < 2.5) solutions.^28^ Theoretical calculations at the MP2/aug-cc-pVTZ and EOM-CCSD/aug-cc-pVDZ level of theory support this hypothesis and predict absorption bands for the various different isomers (see Figure S18) that are consistent with our transient absorption spectra.

The photogenerated adduct formed upon 268 nm excitation is far more prominent when compared to H^+^ quenching experiments with 200 nm (see Figure 3b), where the associated transient features (which we can now assign to H atom adduct signatures) are dwarfed by the indole cation transient. The prominence of the adduct bands gives an indication of the relative distance between the indole cation and the location of the electron scavenged. Thus, H atoms generated from electron scavenging must be on average created at greater internuclear distances from the indole cation at 200 nm than at 268 nm.

TCSPC measurements of acidic indole solutions with 260 nm excitation revealed that the excited state fluorescence lifetime is also shortened by addition of H^+^ (see Figure S12), with a quenching rate constant of 4.45 × 10^9^ M^–1^ s^–1^, a factor of ∼3 smaller compared to literature value for proton scavenging of separated solvated electrons,^63^ and markedly slower than what we observed for quenching-separated solvated electrons generated with 200 nm excitation (see Section 2.2). While the TA data show that protons can scavenge electrons, the TCSPC data demonstrate this also leads to a concomitant reduction in the indole fluorescence lifetime^34^ as per nitrate experiments, implying that the photoproduct containing the electron is closely linked to the parent indole ^1^L_a_ state population.

To explore the fates of the species formed within the first 750 ps into the nanosecond-to-microsecond time regime, nanosecond TA data were acquired with a different instrument in deaerated water solutions using 266 nm excitation with a 5 ns excitation pulse (see the Supporting Information). The data presented in Figure S19 show a transient at 440 nm associated with triplet indole (spectrum shown in Figure 2) as assigned by Bent and Hayon.^28^ The decay of this band is sensitive to oxygen and in deaerated solution has a 15 μs lifetime, in good agreement with Bent and Hayon’s measurements.^28,49^ Importantly, our data between 440 and 450 nm show a rise characterized by 40% further growth with a rise time of ∼100 ns beyond that appearing with the IRF. While we do not have data to show how the absorption in this band evolves after 745 ps, the last slice shown in Figure 4, up until 5 ns, the later rise time observed for the indole triplet far exceeds the ^1^L_a_ state lifetime and therefore cannot derive from the ^1^L_a_ state in a single step, such as conventional ISC following El-Sayed’s rules. There must be additional steps with different rate-limiting steps. Given the nanosecond rise of separated electrons (see Figure 4d) and even slower appearance of the triplet absorption band, we suggest instead that the indole triplet originates from recombination between free indole cations and solvated electrons, as previously hypothesized by Fischer et al.^49^ This ISC mechanism is usually referred to as mediated by radical pair spin–orbit coupling.^74^

For 292 nm, the ^1^L_a_ and ^1^L_b_ electronic states of indole are excited in an approximately equal proportion.^65^ The recorded TA data recorded for this pump wavelength (Figure S20) display a similar spectral profile and time-evolution compared with the data recorded at 268 nm (Figure 4). The estimated ionization thresholds reported by Bernas et al. (285 nm, 4.35 eV)^36^ and Katoh^37^ (290 nm, 4.27 eV) are both slightly above the photon energy associated with 292 nm (4.24 eV) excitation. The kinetics at three probe visible wavelengths are shown in Figure S21 and appear to be very similar to those associated with 268 nm experiments. Scavenging experiments with nitrate and HCl added to solutions showed similar dynamical quenching (see Figures S20 and S22) to 268 nm, and fluorescence quenching efficiency with excitation wavelength does not show a drop off at wavelengths out to ∼290 nm (Figure S23). Therefore, we propose that equivalent photophysical dynamics occur at 292–268 nm. Despite being below the previously reported indole photoionization threshold in water,^36,37^ the channel is accessible at the longer wavelength of 292 nm.

## 3 Discussion

Briefly, to summarize our key observations, the present study indicates that the photoionization channel of indole is entirely solvent dependent—no solvated electrons are observed in ethanol solution at any of the three excitation wavelengths (292, 266, and 200 nm), despite 200 nm being far above the determined ionization potential.^36^ These spectra are therefore dominated by the parent ESA as confirmed by computational data and decay uniformly across all probe wavelengths with the known ^1^L_a_ fluorescence lifetime in ethanol.

The photoexcited indole dynamics in water contrast greatly: 200 nm excitation dominantly ionizes indole in a conventional way via direct ballistic electron ejection into the solvent, with clear signatures apparent of the formation of separated solvated electrons within the 200 nm instrument response (240 fs), which proceeded by a rise in intensity associated with ∼675 nm within the first few picoseconds and associated subsequent blue shifting of a separated solvated electron band. H^+^ quenching experiments confirm the formation of solvated electrons and reveal the known signature of the geminate partner indole cations. TA spectra are far more complex when using 292 or 268 nm pump wavelengths; while a major part of the spectrum is dominated by ^1^L_a_ ESA, there is a small but notable amplitude blue-shift around 600 nm within the first 2 ps, which we associate with the formation of a contact ion-pair which absorbs further to the blue of a separated solvated electron. For 268 nm pumped samples, the kinetics at 730 nm, which is associated with solvated electrons, slowly rise on a nanosecond time scale commensurate to the ^1^L_a_ state fluorescence decay. This slow production of solvated electrons is not without precedent and has been observed in TA studies of phenol and aniline in aqueous solution after excitation of the lowest energy ^1^ππ* state.^40,41^ Experiments with H^+^ and NO_3_^–^ electron scavengers show that both separated electrons and contact pairs are accessible for quenching. For example, in solutions with 0.2 M HCl, there is a reduction in intensity at ∼650 nm, and a characteristic band associated with the cation is evident at later times. The addition of KNO_3_ to indole solutions quenches all species present, including the ^1^L_a_ state of indole, indole cations, and electrons.

Let us now review our results in the context of the rich indole computational literature. Recall that *ab initio* calculations by Domcke and Sobolewski for microsolvated indole–(water)_*n*=1–3_ clusters showed that the ^1^πσ* state is no longer an accurate descriptor as the σ* orbital is not localized to the indole molecule, per the gas phase, but instead “caged” by the surrounding water molecules. The authors instead described the associated excited state as a charge-transfer-to-solvent (CTTS) state. A subsequent nonadiabatic QM/MM surface hopping study by Wohlgemuth et al. of indole with three explicit QM water molecules located around the N–H functional group inside an MM water sphere^75^ showed that surface-hopping trajectories of electron transfer from indole to solvent occurred on a 45 fs time scale. Both studies predicted the electron resides on the water molecule H-bonded to the indole N–H moiety. Wohlgemuth et al. also labeled this as a CTTS state but recognized that within the confines of their limited 3 QM waters, the state is the direct precursor to photoionized products. Gonzalez and co-workers carried out computational studies of the related heteroatomic molecule phenol microsolvated by 15 water molecules, and they described the presence of a charge-transfer (CT) state: an electron promoted from an π orbital on phenol to a σ*-type orbital located in the solvent. The calculations returned very low oscillator strengths for the CT state and showed that the CT minimum is lower in energy than that of the optically bright ^1^ππ* state.^76^ We propose that the aforementioned theoretical discussion of CTTS or CT states in related heteroaromatics molecules is another way of describing, or even synonymous with, a tightly bound cation–electron contact ion-pair,^63,77^ e.g., [Ind^+^:e^–^], where the surfaces of the cation and electron are in direct contact with no solvent molecules in between.

In the context of our 292 and 268 nm indole data, we will now provide an explanation for a tight ion contact pair generation, the species evident within the first 2 ps of our TA experiments, and its relationship with photoionization. TCSPC experiments at 260 nm with protons and nitrate electron quenchers remarkably led to a reduction in the indole fluorescence lifetime. We established with control experiments in ethanol that nitrate cannot directly quench the ^1^L_a_ state of indole. This means that electrons must be scavenged from a species that affects the population of the ^1^L_a_ state of indole, e.g., in equilibrium, and therefore cannot correspond to a spatially separated species such as solvated electrons. As quenching TA experiments in H^+^ showed, this should instead be from a location close to the indole cation geminate partner, and therefore the ^1^L_a_ state of indole must be in a dynamic equilibrium with the tightly bound ion-contact pair, ^1^[Ind^+^:e^–^]. The rise at red-wavelengths in our TA data within the first 2 ps corresponds to either the immediate formation and subsequent solvation of ^1^[Ind^+^:e^–^], or merely a slower formation of the latter. The emergence of ^1^[Ind^+^:e^–^] in our data is in line with QM/MM simulations of indole in water that predicted fast formation of a CTTS state in indole.^75^

Once formed, the ^1^L_a_⇌^1^[Ind^+^:e^–^] equilibrium remains in a quasi-steady state until the ion pair dissociates to generate separated solvated electrons and indole cations. The different kinetics across the entire probed wavelength range therefore reflect the relative absorption of each of the species present and their associated fates, e.g., 450 nm is a wavelength, where the cation and electron bands are expected to make minimal contributions (see Figure 2) and thus the associated kinetics display a decay that most closely resembles (at *t* < 300 ps) the indole fluorescence lifetime. Conversely, kinetics at 730 nm correspond to near the peak of the solvated electron, and thus the kinetics of the e^–^_(aq)_ dominate. 600 and 650 nm probe wavelengths remain static over the 750 ps temporal window because of the overlapping of the ESA signals from the ^1^L_a_⇌^1^[Ind^+^:e^–^] equilibrium, which decay and are replaced with the cation/solvated electron that rises with the same rate.

We propose that the ^1^L_a_ state fluorescence lifetime in water is rate-limited by the dissociation of ^1^[Ind^+^:e^–^] into free radicals because the ionized products are predicted to be energetically “uphill” by 0.2 eV (see Figure S1), and the rise at 730 nm associated with solvated electron production from the dissociation of the ion-pair matches that of the indole fluorescence lifetime. This conclusion is counter to the mechanism suggested by Riedle and co-workers,^25^ who proposed the ionization channel represented a separate and branched pathway of the excited state indole dynamics. Our conclusions are consistent also with the reported temperature-dependent fluorescence quantum yield,^78^ which falls by a factor of 5 upon raising the temperature from 5 to 50 °C in water—the increased thermal energy will push the ^1^L_a_⇌^1^[Ind^+^:e^–^] equilibrium slightly more toward the less thermodynamically favored ion pair. Critically, the same study reports only very small changes in fluorescence quantum yield in methanol solution with temperature, consistent with the absence of the contact ion-pair formation and photoionization pathway in that solvent.

The slow rise of the triplet indole signature (∼110 ns) greatly exceeds the ^1^L_a_ state fluorescence lifetime (4.5 ns, see Figure S10) and thus cannot be generated via a typical ISC mechanism. Based on a mechanism originally hypothesized by Fischer et al.,^49^ we have proposed that the T_1_(^3^L_a_) indole is generated via separation of the ^1^[Ind+:e^–^] contact pair to form free radicals (^2^Ind^+^_(aq)_ and ^2^e^–^_(aq)_), which upon recombination form triplet indole and involve a spin-flip, e.g., radical pair spin–orbit coupling, accounting for the 23–35%^46,47^ triplet quantum yield. This route provides a higher rate to ISC compared to the “typical” ISC mechanism, which is disfavored on the grounds of El-Sayed’s rules. Of course, upon diffusive re-encounter, the two species could also regenerate the ^1^L_a_ state of indole. However, as there is no delayed fluorescence lifetime component (within the 0.01% baseline sensitivity of the TCSPC experiments) associated with regeneration of ^1^L_a_⇌^1^[Ind^+^:e^–^], this implies that spin conversion to ^3^[Ind^+^:e^–^] must be sufficiently energetically “downhill” not to allow back transfer to the fluorescent singlet state.

**Figure 6:**
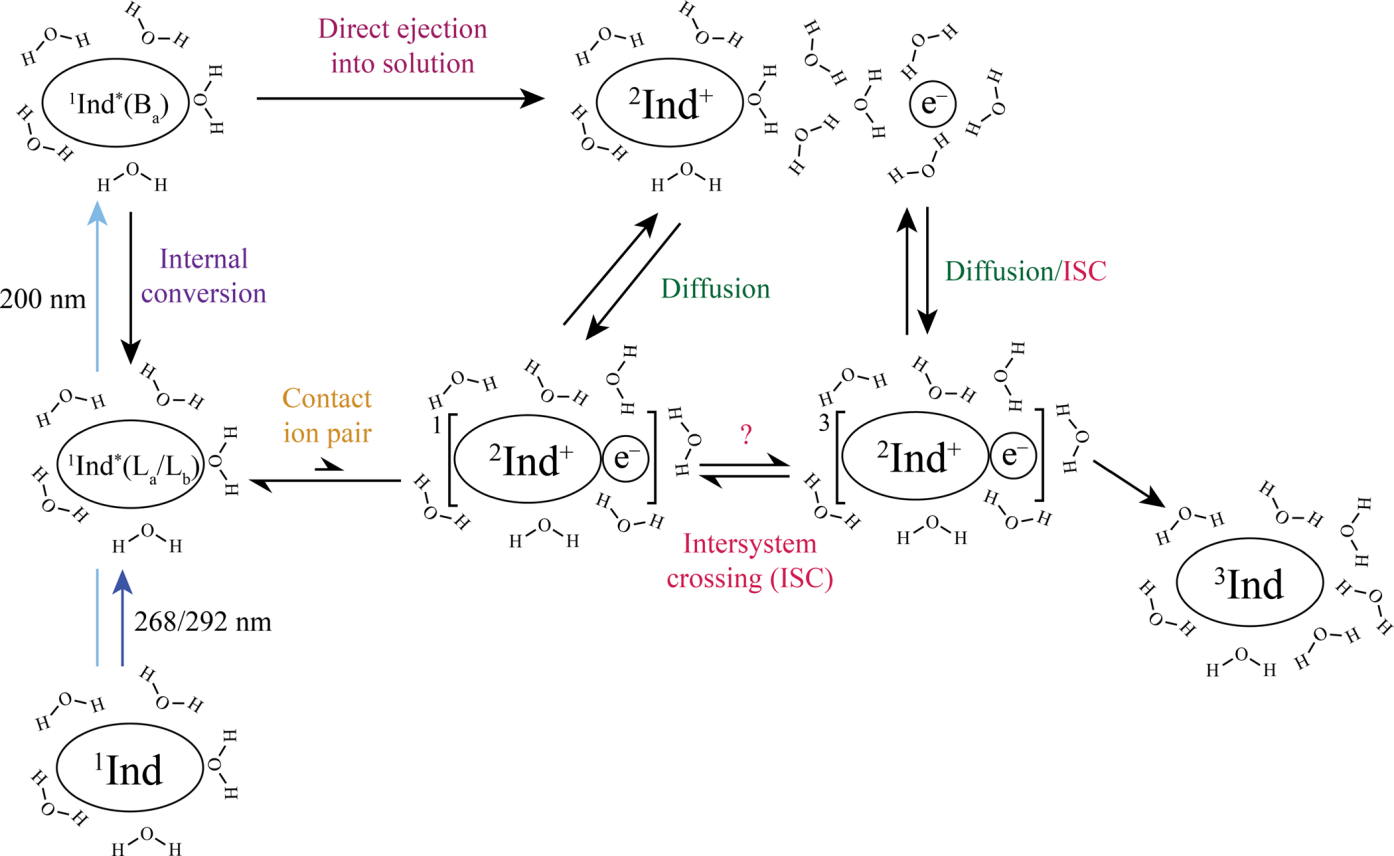
Schematic illustrating the main photoionization pathways of indole (Ind) in aqueous solution and subsequent nonradiative dynamics after 292 and 268 nm compared with 200 nm excitation. Note that diffusion drives the dissociation of the ion-contact pair into charge-separated ions and recombination of the free ions to reform the complex.

Our proposed mechanism for the dominant photoinduced dynamics of indole in water upon 268 or 292 nm excitation is illustrated in the bottom portion of Figure 6.

The dynamics for 200 nm excitation are far less complex; the rise of the electron signal and subsequent solvation is characteristic of electron ejection into the bulk solvent (see upper part of Figure 6), as we observed for phenol with 200 nm excitation.^40^ From our TA data and spectral reconstruction (Figure S8), we conclude that ionization is likely the dominant pathway, reconciling the long-time ionization quantum yield at 193 nm is ∼0.3–0.4,^64^ but is not the sole photochemical pathway at 200 nm, with some competition from internal conversion to populate the ^1^L_a_/^1^L_b_ states.

In the 200 nm TA data, there is also a clear decay phase to the kinetics, where 25% of the intensity at 633 nm has decayed by *t* ∼500 ps (see Figure S6). This originates from initially charge-separated indole cations and solvated electrons (re)-encountering one another to generate the ^1^L_a_⇌^1^[Ind^+^:e^–^] equilibrium, or as a result of full charge-separation the associated spins are able to flip/scramble to form ^3^[Ind^+^:e^–^] and the thermodynamically stable ^3^L_a_ state (0.5 eV more stable than ^1^L_a_ minimum; see Figure S1). Both recombination pathways would generate TA absorption bands in the region 375–500 nm and thus can rationalize the region of the 200 nm TA data at 500 ps that we were unable to reconstruct from Ind^+^_(aq)_ and e^–^_(aq)_ basis functions. Alternatively, recombination could reform the neutral ground state molecule, but we are not sensitive to the ground state bleach kinetics.

Our findings are only partially consistent with the TA studies by Peon et al. using 260 and 262 nm excitation^14,39^ and Bizjak et al. using 270 nm.^25^ One key difference is these prior investigations did not track dynamics over a sufficient range of probe wavelengths and therefore were unable to observe the nanosecond rise of the solvated electron signal at 730 nm and the ^1^L_a_ ESA decay at 450 nm. Both Peon et al. and Bizjak et al. proposed that indole photoionization occurred on an ultrafast time scale, with Peon et al. noting a lack of rise on a 100 fs to 2 ps time scale expected for electron solvation at 720 nm—an observation in line with our data. In contrast, we have shown that ionization is not immediate and instead, the ^1^L_a_ state of indole is in equilibrium with ^1^[Ind^+^:e^–^] that slowly dissociates on a nanosecond time scale to generate the separated photoionized indole cations and solvated electrons products. Bizjak et al.^25^ also showed a significant signal intensity rise at 650 nm (∼50% amplitude) within <1 ps and associated this with prompt solvated electron production. The large amplitude rise contrasts with data presented by Peon et al. and the present study (see Figure 4). We surmise that this must originate from multiphoton solvent ionization and the origin of the reported instantaneous 45% ionization yield. Kohler’s group argued that there was no geminate recombination with 262/260 nm excitation, which we agree is the case within their 100s ps time window,^14,39^ but for a different reason: only a small fraction of indole molecules fully photoionize in this time range. After the ion-pair dissociation, we observe some geminate recombination by triplet indole production. Further, counter to Peon’s arguments about a large barrier to recombination, in our 200 nm experiments in aqueous solution, promptly ionized products do indeed recombine (∼25% within 500 ps).

As previously highlighted, our TA data indicate the photoionization channel in indole is accessible at 292 nm in water and thus below the previously reported ionization threshold in water.^36,37^ 292 nm lies on the rising red edge of the first vibronically resolved feature in the steady-state absorption spectrum. Notably, the absorption spectrum has a long wavelength tail that extends down to >300 nm (see Figure S2). Steady-state fluorescence excitation experiments with protons revealed that H^+^ quenching efficiency was invariant between 260 ≤ λ ≤ 290 nm (Figure S23), implying that the ionization quantum yield is static across this wavelength range and insensitive to internal vibrational excitation.

The photoionization of indole seems to be unique to water, as ionization is not observed in ethanol with any excitation wavelength, despite 200 nm being above the predicted ionization threshold.^36^ Similarly, transient studies in other alcohols such as methanol (see Figure S24 and ref (32)) and 1-propanol^14,39^ exciting the ^1^L_b_/^1^L_a_ states at ≥260 nm also show no signature of indole photoionization. We speculate that this channel is only evident in water because the greater polarity of the solvent greatly stabilizes the ionized products (the dielectric constant of water is >2× that of methanol), as will the very strong hydrogen bonding between solute and solvent evident in water deduced from ab initio modeling of recent XPS experiments.^60^

## 4 Conclusions

We have shown that the photoionization mechanism of indole in water has a marked wavelength dependence: at 200 nm, ejections are ballistically ejected into bulk solution on an ultrafast time scale. In contrast, with 268 and 292 nm excitation, photoionization takes nanoseconds to occur, requiring the dissociation of a promptly formed tightly bound ion-contact pair. Our results show that the ionized products are key precursors to triplet indole formation and dramatically increase the rate of ISC.

The photochemical dynamics of indole in aqueous solution presented here is far more representative of a solvent-exposed tryptophan residue in a protein than isolated tryptophan in water. At pH 7, the amide group of the tryptophan in water is zwitterionic, and the protonated ammonium group is the source of an intramolecular proton transfer to the aromatic ring, which leads to quenching of photoexcited tryptophan.^79,80^ The TA data of tryptophan in a peptide (NCp7)^79^ show remarkable spectral and temporal similarities to our 268 nm data of indole in solution, indicating that our detailed mechanism is likely the most appropriate for describing photodynamics of tryptophan in its native environment.

Our photophysical model is able to rationalize the environmental sensitivity of the indole side moiety to water-rich environments, which strongly modulates the fluorescence quantum yield–an important property that is regularly used by biochemists and biophysicists to probe whether tryptophan residues are solvent exposed, or buried inside the protein.^2^

## Data Availability

The data underlying this study are openly available in Zenodo at https://zenodo.org/doi/10.5281/zenodo.10933429.

## Supporting Information Available

Experimental and theoretical methods; steady state characterization of indole in water and ethanol; TA slices of ethanol data; additional 200 nm TA data and spectral reconstruction; time-correlated single photon counting at 260 nm; comparison of kinetics acquired for 268 nm pump wavelengths; nanosecond 266 nm TA data; calculated adduct structures and associated energies; and 292 nm transient absorption data in water (PDF)
