# Supplementary material for: Unraveling the Photoionization Dynamics of Indole in Aqueous and Ethanol Solutions
Source: J Phys Chem B. 2024 Apr 24;128(17):4158–70. doi: 10.1021/acs.jpcb.4c01223 (PMC11075084; doi:10.1021/acs.jpcb.4c01223)
Supplement: Supplementary file 1 — jp4c01223_si_001.pdf [file jp4c01223_si_001.pdf]

Electronic Supplemental Information for:

Unravelling the Photoionization Dynamics of Indole in Aqueous and Ethanol Solutions

Gaurav Kumar,[1†] Michael Kellogg,[1] Shivalee Dey,[1]

Thomas A. A. Oliver,[2*] and Stephen E. Bradforth[1*]

[1]*Department of Chemistry, University of Southern California, Los Angeles CA 90089, United States*

[2]*School of Chemistry, Cantock's Close, University of Bristol, BS8 1TS, United Kingdom*

Current address: †*Intel Corporation, NE Butler St, Hillsboro, OR, 97124, United States*

1. Experimental and Computational Methods

Indole (99%; Sigma Aldrich) was purchased and used without further purification. The steady state UV absorption spectrum of indole (50 μM) in water and ethanol was recorded using the Cary 50 UV-Visible spectrophotometer while the steady state fluorescence measurements were carried out using a fluorimeter (Jobin Yvon Fluoromax 3) and a 1 mm path length cuvette.

Transient absorption experiments were performed using the output of a 35 fs Ti:Sapphire amplified laser system (Coherent Legend Elite USP, 1 kHz repetition rate). These experiments were performed by exciting solutions of indole with UV pulses (λ_{pump} = 200, 266, 268 and 292 nm) and probed using white light super-continuum pulses. To generate 200 nm pump pulses, a portion of the Ti:Sapphire laser fundamental (800 nm) was gently focused by a *f* = 2 m lens into a 500 μm *type*-I β-barium borate (BBO) crystal (Red Optronics) to generate 400 nm. The resulting 400 nm laser pulses were used to generate 267 nm by sum frequency mixing of 400 nm with a part of the residual 800 nm in a 150 μm *type*-II BBO crystal (Red Optronics). The 267 nm had a bandwidth

of 3–4 nm with a maximum pulse energy of 8–9 μJ and was used again to generate 200 nm (1 μJ) via sum frequency generation with the residual 800 nm (60 μJ) in a 75 μm thick *type*-I BBO crystal (Red Optronics), which was placed at the focus of the 267 nm and the 800 nm. The deconvoluted temporal width of the 200 nm was ~220 fs, as determined by the cross-correlation with the probe continuum in ethanol solution.

The other pump wavelengths 266, 268 and 292 nm (with typical bandwidths of 4.5–5 nm) were generated by doubling (using a 150 μm *type*-I BBO crystal (Red Optronics)) the visible output from a homemade Non-Collinear Optical Parametric Amplifier (NOPA) following the design of Riedle *et al.*[1] To achieve the optimal temporal resolution in our experimental set-up, the visible output and the second harmonic of the NOPA output were optimized using prism-pair compressors, with prisms of fused silica and calcium fluoride respectively. The deconvoluted temporal width of the 266 nm was determined by the cross-correlation with the continuum in ethanol solution to be ~70 fs. 266 nm pulses were used for experiments in ethanol, whereas 268 nm was used for data acquired in aqueous solution.

The pump beam diameter was measured to be ~160 μm at the sample focus, with associated 1000, 260 and 280 nJ pulse energies for experiments at 200, 268/266 and 292 nm.

The probe continuum broadband, ranging from 320 nm to 700 nm, was produced by focusing a small fraction of 800 nm fundamental onto a rotating calcium fluoride window (2 mm thick, Koch Crystal Finishing). A pair of aluminum-coated off-axis parabolic mirrors (Janos Technology) were used to first collimate the resulting supercontinuum beam and second to focus the probe into the sample. The relative polarization between the pump and probe pulses was controlled by rotating the 800 nm polarization prior to continuum generation with an air-spaced zero-order half waveplate (Karl Lambrecht Corporation). All the experiments reported here were performed at a magic angle between the pump and probe pulses. In all cases, the signal arising for the solvent alone, with the same pulse energy and spot size, was carefully checked. The induced absorbance from solvated electrons arising from solvent ionization is never more than 5% of the signal due to indole photoexcitation.

Aqueous indole (10–20 mM) was flowed through a recirculating wire-guided gravity jet which produced a thin film of the liquid with thickness of 115 μm.[2] The exact path length of the sample varies depending upon the liquid property such as density, viscosity, and the distance of the

interaction region from the nozzle opening. A variety of advantages are realized when using liquid film to carry out experiments: (a) to minimize the group velocity walk-off between the deep-UV pump and the broadband continuum, (b) avoid the contamination of the TA signal from non-resonant coherent signals (such as two-photon absorption) from the quartz/glass (of a cuvette) and (c) to ensure a new sample volume is presented at the laser interaction region for every laser shot, which ensures no signal is recorded that corresponds to photo-degraded sample. The dispersion in the TA signals were corrected by measuring the signal from the pure solvent under the same experimental conditions and adjusted during data analysis by interpolation with a third order polynomial.

Fluorescence lifetimes were measured using time-correlated single photon counting (TCSPC). An ~ 100 fs pump pulse centered at 260 nm is generated by frequency-doubling a Coherent OPA 9450 optical parametric amplifier tuned to 520 nm that is driven by a Ti:sapphire regenerative amplifier (Coherent RegA 9050, 800 nm) operating at 100 kHz repetition rate. The 260 nm excitation pulses, which are polarized parallel to the laser table, are focused on the sample with a focusing lens of 10 cm. Excitation pulse energies in the range of 0.3 – 0.45 nJ were utilized with a spot size of 45 ± 2 μm (FWHM). The emission wavelength detected was set at 360 nm; emission is collected with a 1-inch lens in a perpendicular geometry to the excitation beam and refocused on the entrance slit of a Digikröm CM112 double monochromator set out to preserve time resolution. The emission bandwidth detected is 4.5 nm using slits throughout the monochromator of width 0.6 mm. A Hamamatsu R3809U-50 PMT attached at the exit slit of the monochromator was operated at 3 kV. The signal is recorded by a Becker and Hickl SPC-630 time-correlated single photon counting instrument. To ensure pulse pile-up effects do not distort the decay lifetime, all the experiments were performed with photon counting rates less than 2% of the repetition rate of the laser. The instrument response of the experiment was measured using scattered pump light (260 nm) and determined to be 22 ps. Experimental data are fit to mono- or bi- exponential decays.

For the measurements, 17 μM of indole (> 99%, Sigma Aldrich without further purification) dissolved in distilled H_2O was used for all the TCSPC experiments. This corresponds to an optical density of 0.15 OD in 1 cm quartz cuvette at 260 nm. Solutions were made up with potassium chloride or potassium nitrate (VWR, >99%); the nitrate anion serving as a quencher.

Nanosecond to microsecond transient absorption data was recorded using a Magnitude enVISion system equipped with an external 532 nm Nd:YAG laser that is frequency doubled to 266 nm and after an optical chopper illuminates the sample at a repetition rate of 1 kHz. ~ 70 μJ of 266 nm is gently focused to a ~ 5 × 2 mm oval and overlapped with the light from a xenon lamp in a 1 × 0.5 cm flow cell where 1L of aqueous de-aerated indole solution is continuously flowed to avoid sample degradation. A monochromator is used to select single probe wavelengths (10 nm bandwidth) from the white light transmitted through the cell and a fast photodiode reads out the time response of the transmitted beam. An oscilloscope digitizes the signal, and the transient absorbance is computed. The instrument response, determined by the laser pulse width and the fast photodiode response is ~5 ns. The kinetics of the triplet absorption is captured by recording the band at 440 – 450 nm.

All theoretical calculations were all performed using Molpro 2015.1,[3-4] and performed on a single isolated indole molecule. The ground state molecular structure of indole was optimized with MP2 using Dunning's aug-cc-pVTZ basis set. In the gas phase the ^1L_b minimum is lower in energy and the lowest excited single state (S_1), however, for consistency with the experiments in solution where the ^1L_b/^1L_a state ordering inverts at $t > 5$ ps, S_1 is referred to the ^1L_a state, and S_2 as the ^1L_b state also when discussing our computational results. The S_1 and S_2 excited state geometries were optimized using the CASSCF method and a 10 e^- in 9 orbitals active space and the aug-cc-pVTZ basis set. The ground state molecular geometries of the indole radical and cations were optimized using CASSCF and 9/10 and 9/9 active spaces, respectively, both with an aug-cc-pVDZ basis set. The vertical excitation energies for the lowest lying states for the neutral molecule, cation and radical were computed at the CASPT2 level using the same active space and an aug-cc-pVDZ basis. The $S_n \leftarrow S_1$ and $S_n \leftarrow S_2$ excitation energies and associated transition dipole moments of neutral indole were calculated with EOM-CCSD using an aug-cc-pVTZ basis set. The product of a reactions between indole cations and H atoms (henceforth referred to as adducts) were characterized by MP2/aug-cc-pVTZ calculations, and the associated electronic absorption bands calculated at the EOM-CCSD/aug-pVDZ level of theory.

2. Energy Landscape for Isolated and Solvated Indole

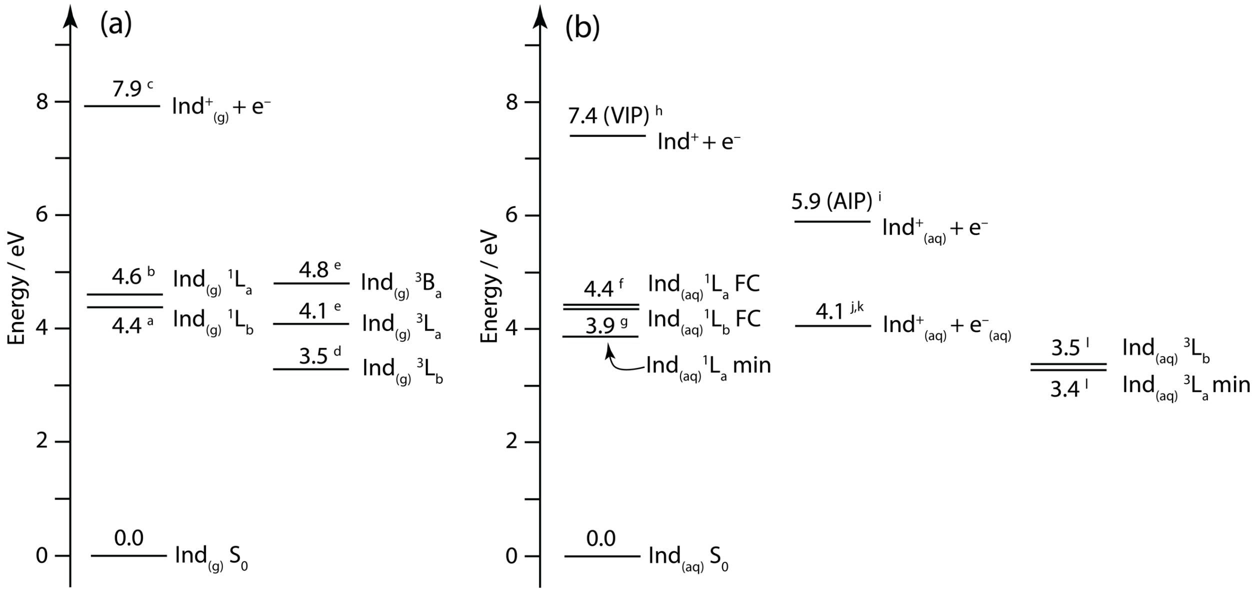

Figure S1. Energy landscape for indole and associated photoproducts in (a) gas phase, (b) aqueous solution where Ind = indole. The energy of each state is labelled in eV next to the horizontal line.

[a] Gas phase ^1L_b origin: 35231.4 cm^{-1}.[5]

[b] Gas phase predicted ^1L_a origin: 37078.4 cm^{-1}.[6]

[c] Gas phase vertical ionization potential.[7-8]

[d] CASPT2 calculated values.[9]

[e] Calculated energy gap from first triplet state at CASPT2 level.[9]

[f] The ^1L_b and ^1L_a electronic origins in the Franck-Condon region are estimated from steady state absorption measurements from this work (Fig. S2), and returned at 285 (4.35 eV) and 280 nm (4.4 eV).

[g] Energetic ordering of ^1L_b and ^1L_a states invert after excited state solvation. The ^1L_a state becomes the lowest energy state. The ^1L_a solvated minimum is estimated from the ^1L_b origin and subtracting the dynamic Stokes shift (3800 cm^{-1}) reported for Tryptophan in water.[10]

[h] Vertical ionization potential (VIP) obtained from liquid jet R2PI measurements,[11] and X-ray PES study.[12]

[i] VIP for indole aqueous indole within experimental error of tryptophan measured with XUV radiation. Adiabatic ionization potential expected to be very similar and reported from the onset of electron formation (5.9 eV) using tunable XUV radiation.[13]

[j] Stabilization of –1.54 eV for solvation of electron[14], and –0.3 eV for correction of the conduction band origin.[15]

[k] Energy of ion contact pair is within k_BT of the separated ions due to the balance in solvation energies of the independent particles compared to the Coulombic attraction inside the ion- (yet overall neutral) pair.

[l] First peak in the 77K phosphorescence spectrum (406 nm (3.1 eV) in MeOH, 404 nm (3.1 eV) in EtOH)[16] likely under-estimates the true T_1 origin in solution. If the Stokes shift for the respective singlet states is included, the T_1[12] energy is estimated to be 3.57 eV. A second approach using the CASPT2 gas phase calculations for the triplet states[9] and shifted according to the experimentally determined water solvation energy for the respective singlet L_a and L_b states yielded a similar T_1 state energy.

3. Steady state absorption and fluorescence

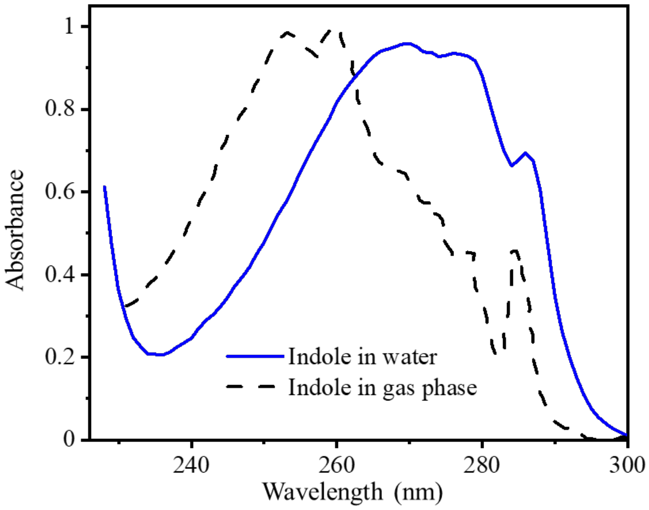

Figure S2. Normalized UV absorption spectra of indole in water and the gas phase.

The steady state excitation and emission spectra of indole were measured in water and ethanol (Fig. S2). The unstructured fluorescence spectra from excitation with 266 nm and 292 nm are both attributed to emission from the ^1L_a state, the fluorescent state in polar solvents as opposed to from the ^1L_b state as observed in non-polar solvents.[9-10, 17] The ^1L_a state fluorescence is strongly solvatochromatic even between ethanol and water due to the large permanent dipole moment of the ^1L_a state. Since water is more polar than ethanol, the ^1L_a stabilization is more substantial in water, leading to a 25–30 nm increased Stokes shift compared to ethanol (Figs. S3(c,d)). This observation is consistent with the reported literature values explaining the state reversal of ^1L_a and ^1L_b which occurs in polar solvents.[9, 17]

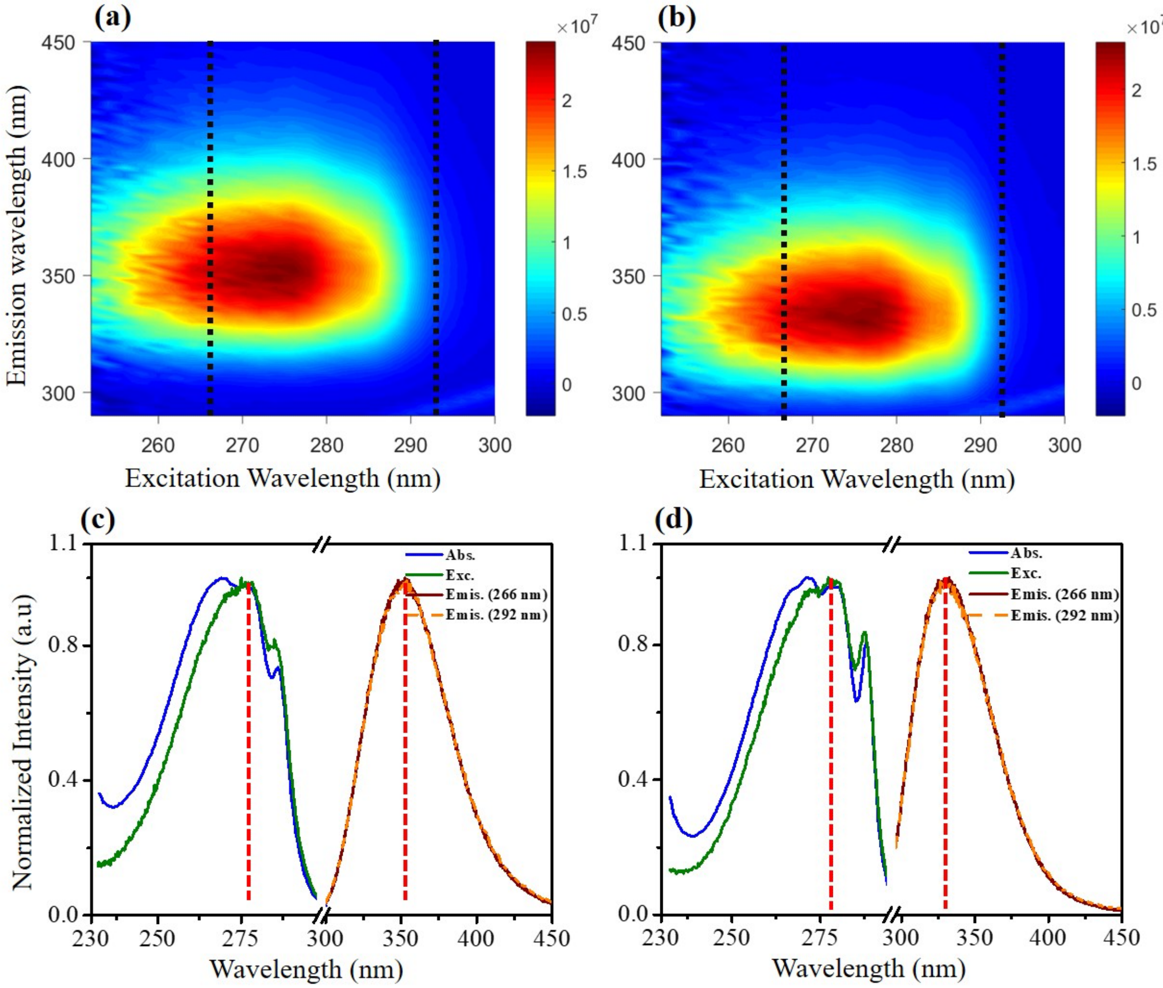

Figure S3. 2D steady state fluorescence spectra of indole (50 μM) in (a) water and in (b) ethanol. Excitation and emission spectra for indole in (c) water and in (d) ethanol. The overlaid red dashed lines indicate the absorption and fluorescence maxima. The pump wavelengths used for time resolved experiments are marked by the black dotted line in 2D plots and the color bar is in units of counts.

4. Transient Absorption Data of Indole in Ethanol

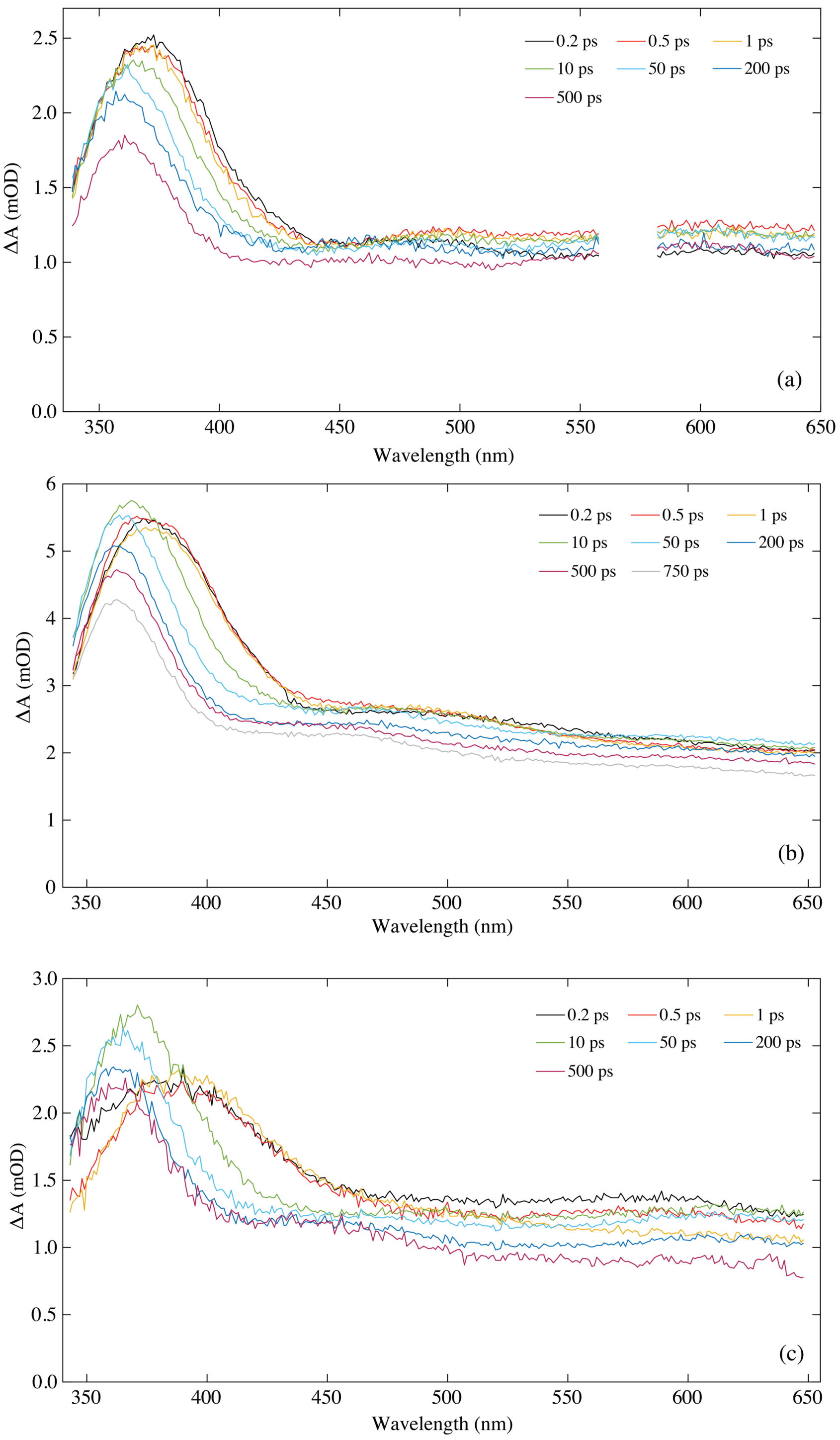

Figure S4. TA spectra for indole in ethanol using (a) 292 nm, (b) 266 nm and (c) 200 nm pump wavelengths. Missing region in (a) due to harmonic of pump scatter.

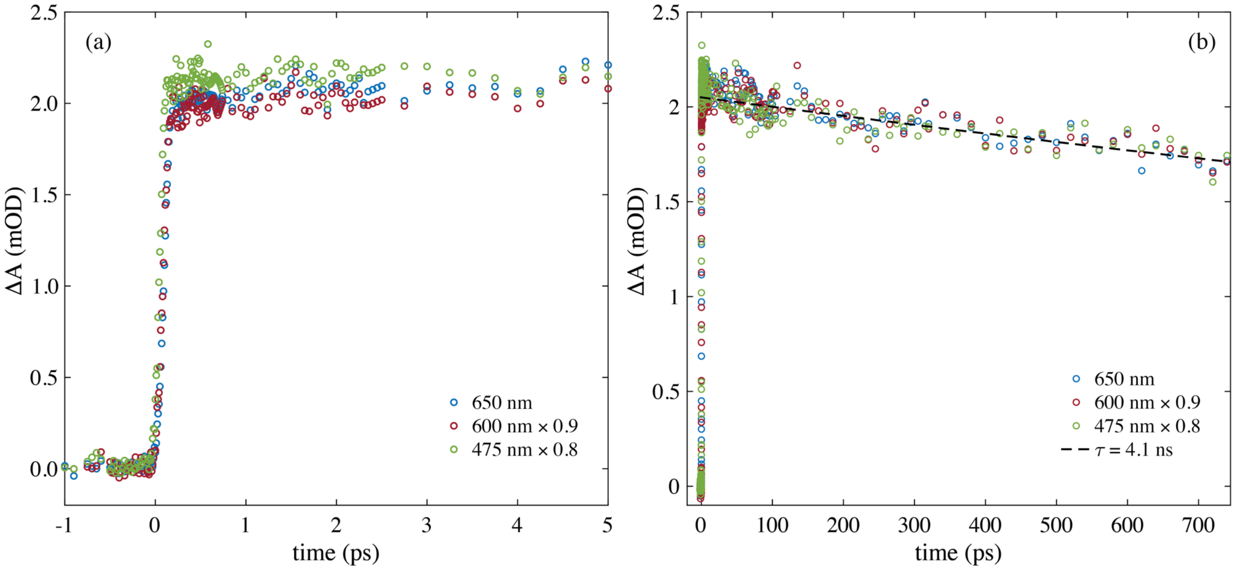

Figure S5. Kinetics associated with 650, 600 and 475 nm of indole dissolved in ethanol pumped at 266 nm displayed for two time ranges: (a) $-1 \leq t \leq 5$ ps and (b) up to 750 ps. Kinetics have been scaled to match those at 650 nm at $t > 50$ ps. Note that the hundreds of fs slow rise observed at 650 nm is also evident in the kinetics associated with 600 nm. Data at 475 nm do not show a marked slow rise outside of the instrument response, inline with recent *ab initio* non-equilibrium calculations which do not predict any marked spectral shifting of the S_1 ESA associated with excited state solvation.[18] Dashed line in panel (b) illustrates exponential decay of 4.1 ns, associated with the ^1L_a fluorescence lifetime of indole in ethanol from Gryczynski *et al.,*[19] and precisely matches the TA kinetics for the three displayed probe wavelengths.

4. 200 nm Transient Absorption Spectroscopy

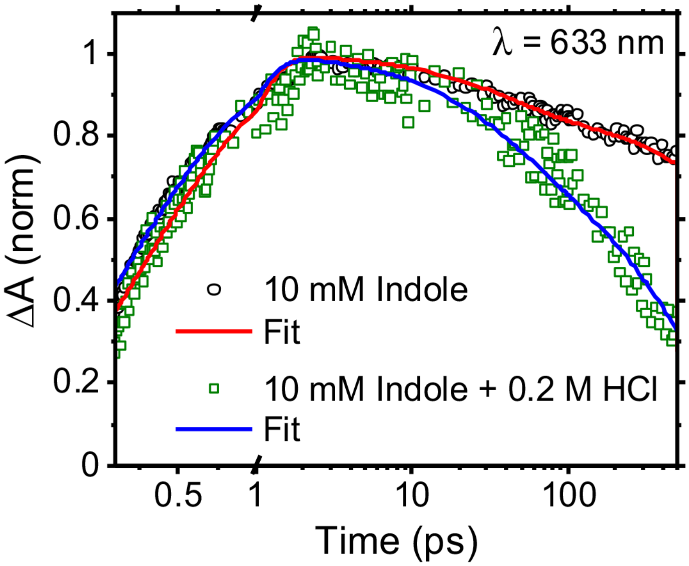

Figure S6. Kinetics recorded at 633 nm for aqueous indole with and without HCl. Note that the time-axis is displayed on a split linear-log scale.

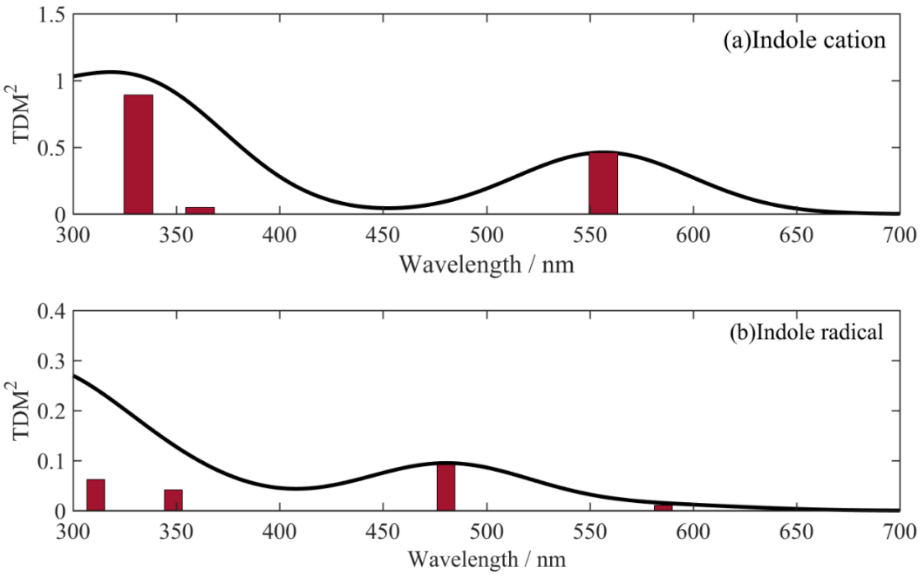

Figure S7. Computed spectrum of indole (a) cation and (b) radical in the gas phase at CASPT2/aug-cc-pVDZ level of theory.

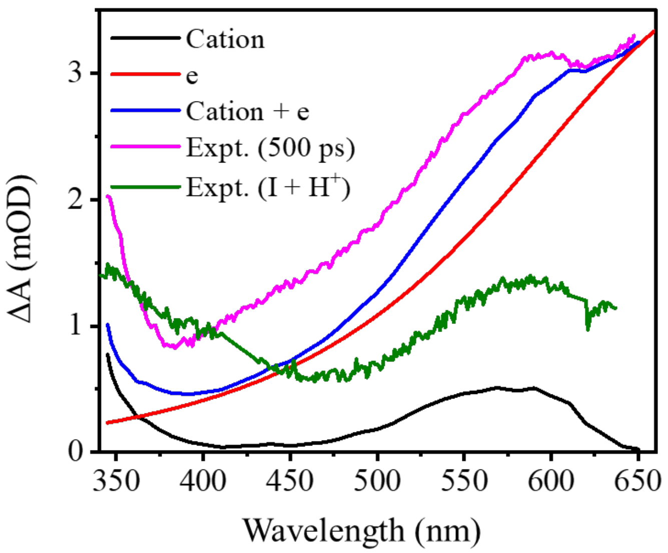

Figure S8. Comparison between the experimentally measured spectrum of 16 mM indole in water with 200 nm excitation and 500 ps time delay, spectra associated with photogenerated species and synthesized spectrum by adding the reported spectra of solvated electron and the indole cation (Fig. 2 main text). The ratio of solvated electron and cation was kept constant while the spectrum was constructed, and a multiplicative factor was used to scale the spectra of solvated electron and cation before summation to produce the constructed spectrum (Indole cation + e^{-}_{aq}). The experimentally measured spectrum of acid-quenched indole in water at long delay (500 ps) is also overlaid - green curve.

Calculated Excited State Absorption Spectra

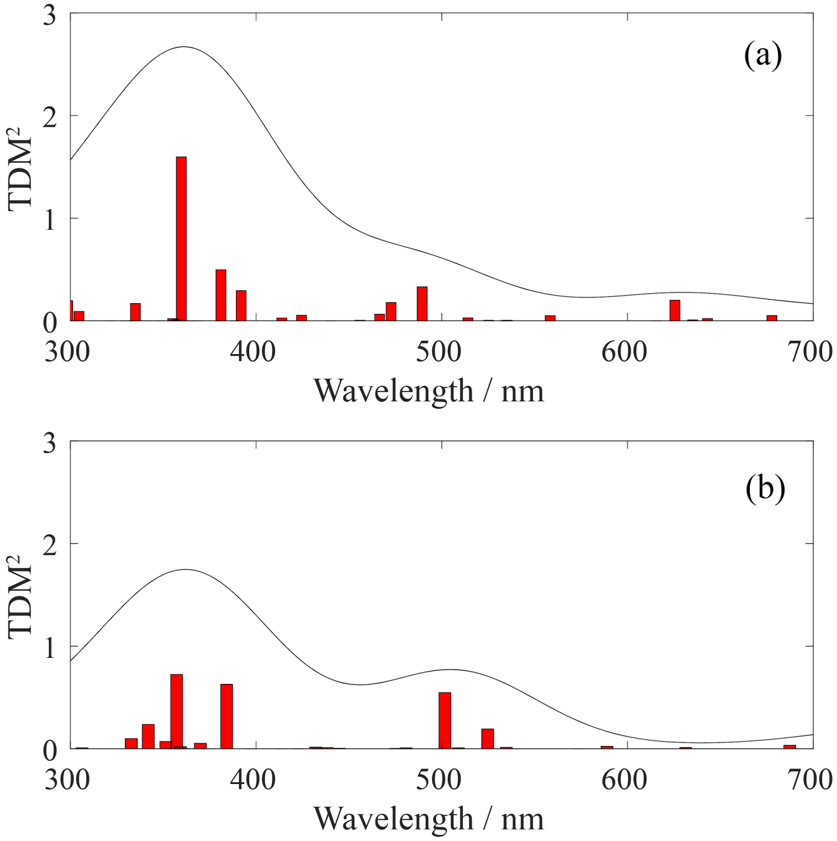

Figure S9. Calculated gas phase EOM-CCSD/aug-cc-VDZ excited state absorption spectra of the indole (a) ^1L_b and (b) ^1L_a states.

5. 260 nm TCSPC KNO_3 and HCl quenching

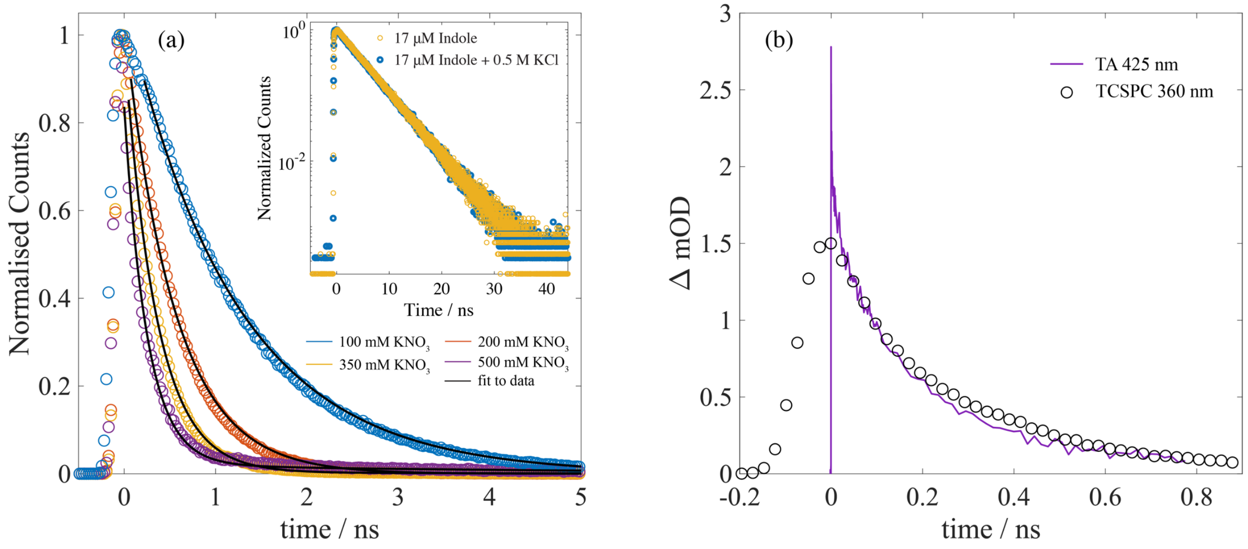

Figure S10. (a) 260 nm TCSPC data of 17 μM indole in aqueous solutions with varying KNO_3 concentrations for 360 nm detection. Inset shows the full fluorescence lifetime data without quencher, which is well fit by a single exponential with $\tau_f = 4.562 \pm 0.004$ ns for pure water; (b) Comparison of time-resolved fluorescence (17 μM indole) and transient absorption data (10 mM indole) for aqueous solutions with 0.5 M KNO_3 added for a probe wavelength where indole ^1L_a ESA signal dominates. TCSPC data were scaled to match the kinetics of the TA data at $t > 0.1$ ns.

Fluorescence lifetime experiments carried out in the presence of the same quenchers as used in the transient absorption experiments in the main paper served to be instructive. Time-resolved fluorescence data recorded at 260 nm are shown in Fig. S10 for indole in aqueous solution. All data are fitted and analyzed by fitting to either a single or double exponential function.

The inset of Fig. S10(a) shows the fluorescence decay for aqueous indole (without quencher) is well described by a single exponential, recovering a lifetime in good agreement with the literature,[20] $\tau_f = 1/k_{ind}$. The fluorescence lifetime for aqueous indole in water did not vary with ionic strength as evident by the overlaid 0.5 M KCl trace.

The series of nitrate concentration solutions show progressively faster fluorescence decay, consistent with the excited state being diffusively quenched. The nitrate data were fit to:

$$I = A_1 e^{-k_{ind} t} + A_2 e^{-k_{obs} t}, \qquad \text{(S2)}$$

where k_{ind} was constrained to the value determined in pure water, and k_{obs} related to the bimolecular quenching rate (k_{quench}) constant *via*: $k_{obs} = k_{quench} [NO_3^-] + k_{ind}$. Figs. S11(a-b) shows the nitrate

concentration dependence of k_{obs}, as well as an observation of relatively little variation in the quenching rate as a function of ionic strength.

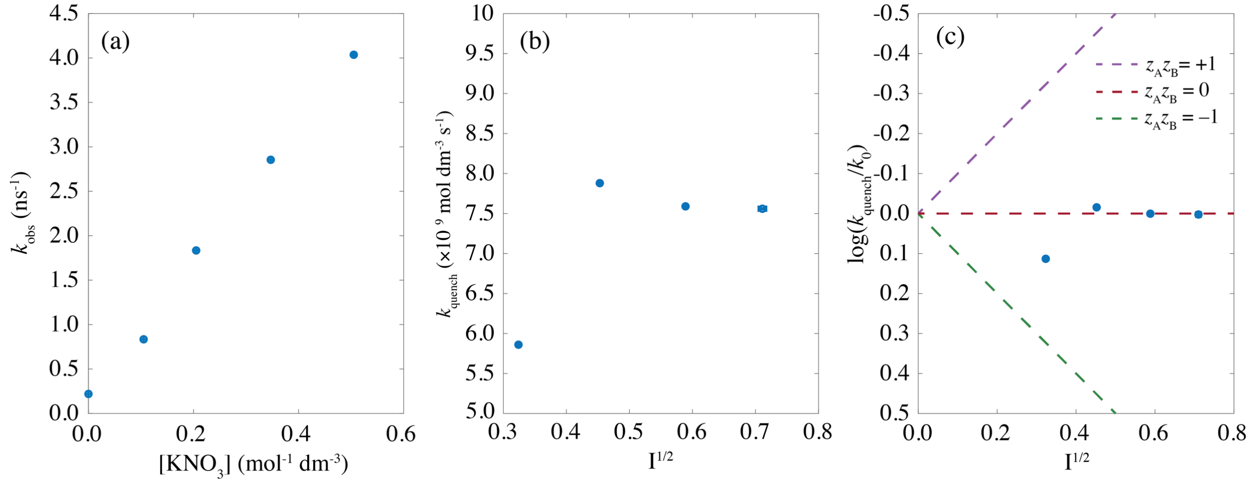

Figure S11. Nitrate quenching analysis (a) k_{obs} as function of nitrate concentration, (b) k_{quench} as a function of square root of ionic strength, and (c) Brønsted-Bjerrum analysis- overlaid dashed lines denote the limiting slopes predicted by Debye-Hückel theory for three different reactive charge states of the two species z_A and z_B. Note data points are plotted as open circles and error bars are smaller than most data points.

A Brønsted-Bjerrum analysis (Fig. S11(c)) again shows no strong ionic strength dependence, and implies the transition state has a single negative charge (*e.g.* NO_3^-), supporting the notion that the electron scavenged throughout the reactive process is tied up with the indole cation in a contact pair (overall neutral charge), rather than with a free solvated electron (which would follow the purple dashed line corresponding to $z_A z_B = +1$). As the nitrate quenches the vast majority of the indole fluorescence, it also means that the contact pair must be in equilibrium with the ^1L_a state of indole. The bimolecular nitrate quenching rate constant was estimated to be 7.6×10^9 M^{-1} s^{-1}, which is comparable to the nitrate quenching rate constant of solvated electrons at infinite dilution (9.7×10^9 M^{-1} s^{-1}).[21]

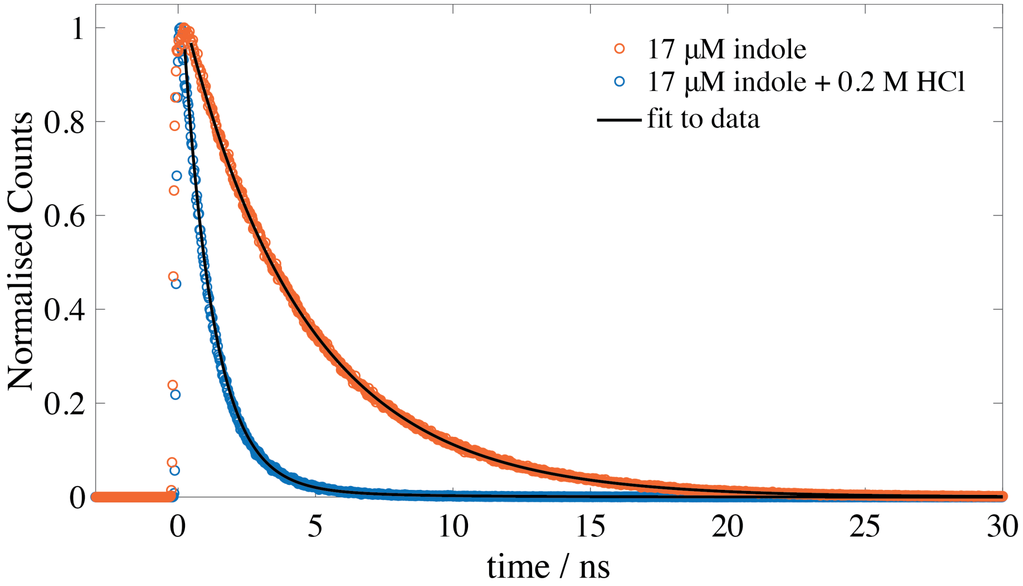

Figure S12. Aqueous indole fluorescence quenched with 0.2 M H^+ compared to indole in pure water, with fits described in the text.

A similar experiment to that carried out in Fig. 5 in the main manuscript using 0.2 M HCl was carried out monitoring the indole ^1L_a state fluorescence. The fitted data with HCl returns $k_{quench} = 4.45 \times 10^9$ M^{-1} s^{-1} which is about a third of the reported quenching rate constant, 1.3×10^{10} M^{-1} s^{-1}, for electrons scavenged at $[H^+] = 0.2$ M

TCSPC experiments of indole dissolved in ethanol were used as a control measurement, as indole does not photoionize in this solvent (see Fig. S13). These data are unlike those in water (see Fig. S10) and show no change in the fluorescence lifetime of indole upon addition of KNO_3, and thus confirming nitrate is unable to *directly* quench ^1L_a indole.

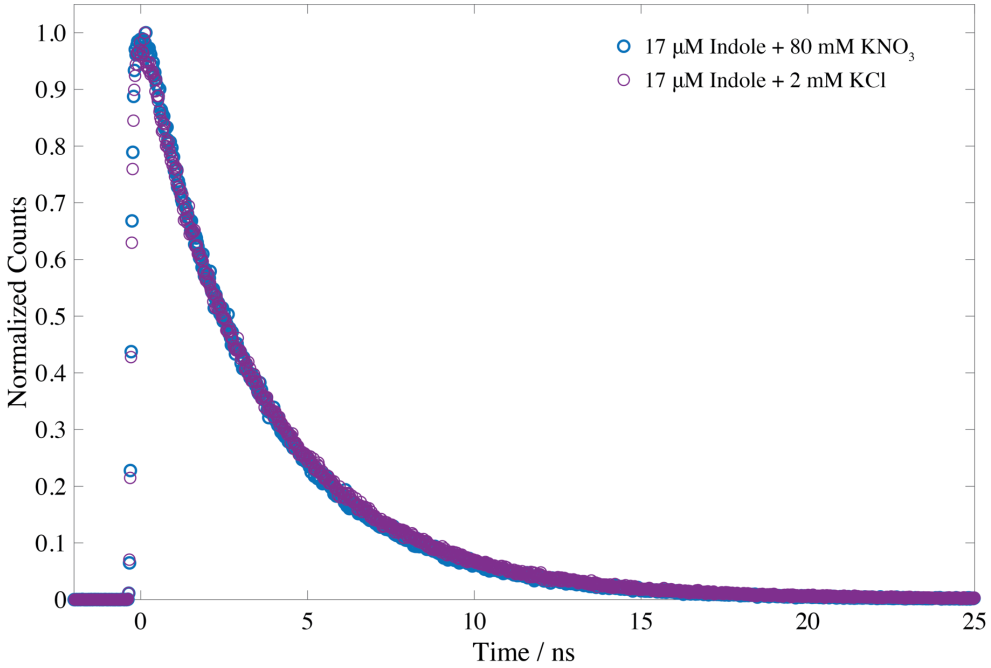

Figure S13. Ethanol TCSPC experiments comparing 80 mM KNO_3 with 2 mM KCl (concentrations of salts limited by low solubility in ethanol).

6. 268 nm Aqueous Indole Transient Absorption

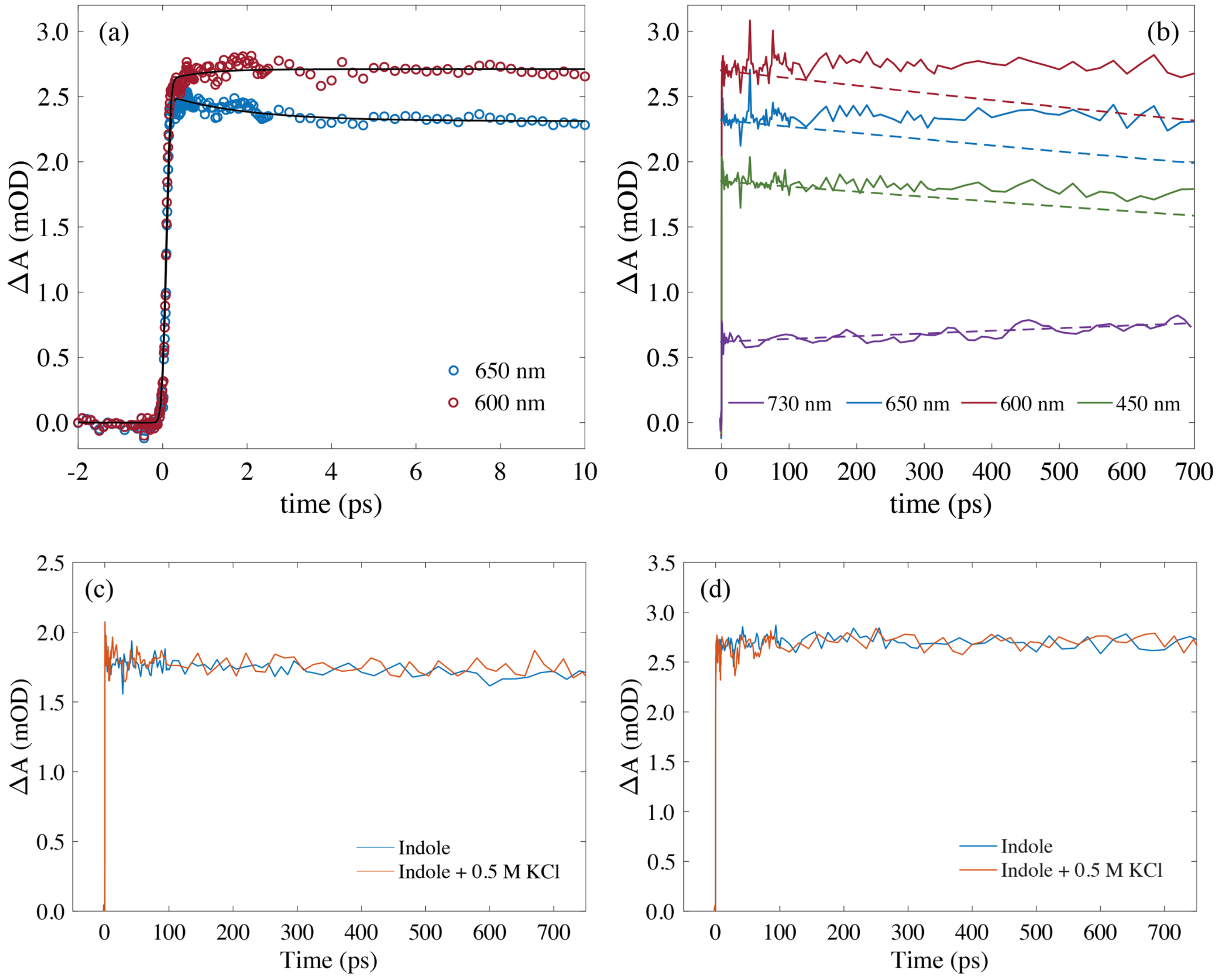

Figure S14. Kinetics for shown probe wavelengths after photoexcitation of indole in water with 268 nm for (a) early time delays and (b) full kinetic range. Effect of 0.5 M KCl on indole kinetics in aqueous solutions for probe wavelengths (c) 440 nm and (d) 575 nm upon 268 nm excitation. Data with KCl salt was scaled to match data in water.

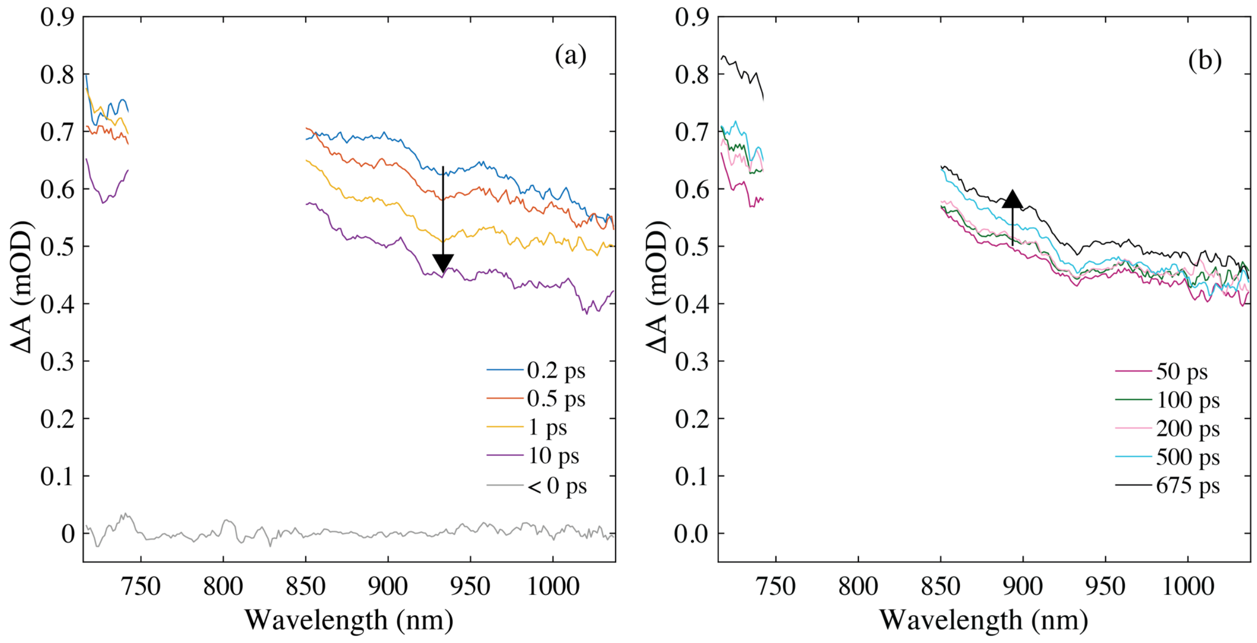

Figure S15. Spectral slices for the aqueous indole (5 mM) for 268 nm excitation displaying the near-IR probe region.

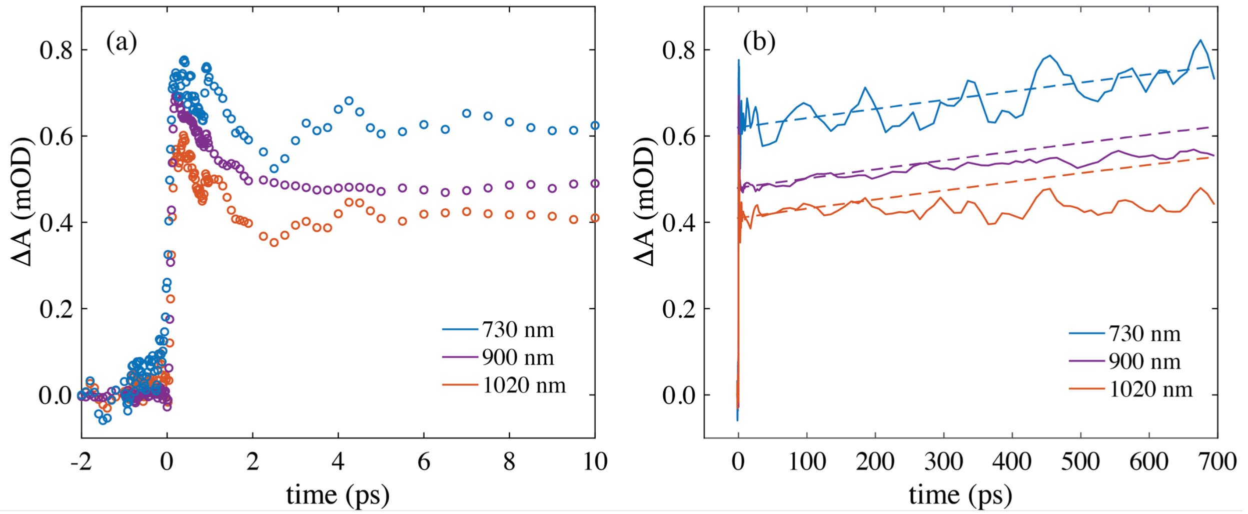

Figure S16. Kinetics for three different near-IR probe wavelengths of indole in water after 268 nm excitation.

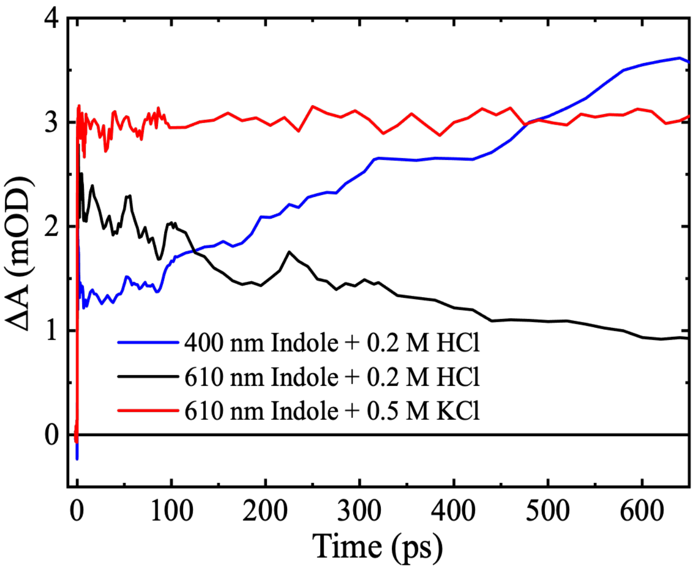

Figure S17. Kinetics for indole excited with 268 nm, comparing the kinetics upon addition of KCl (control) and HCl (electron scavengers) to aqueous 10 mM solutions.

7. Adduct Calculation

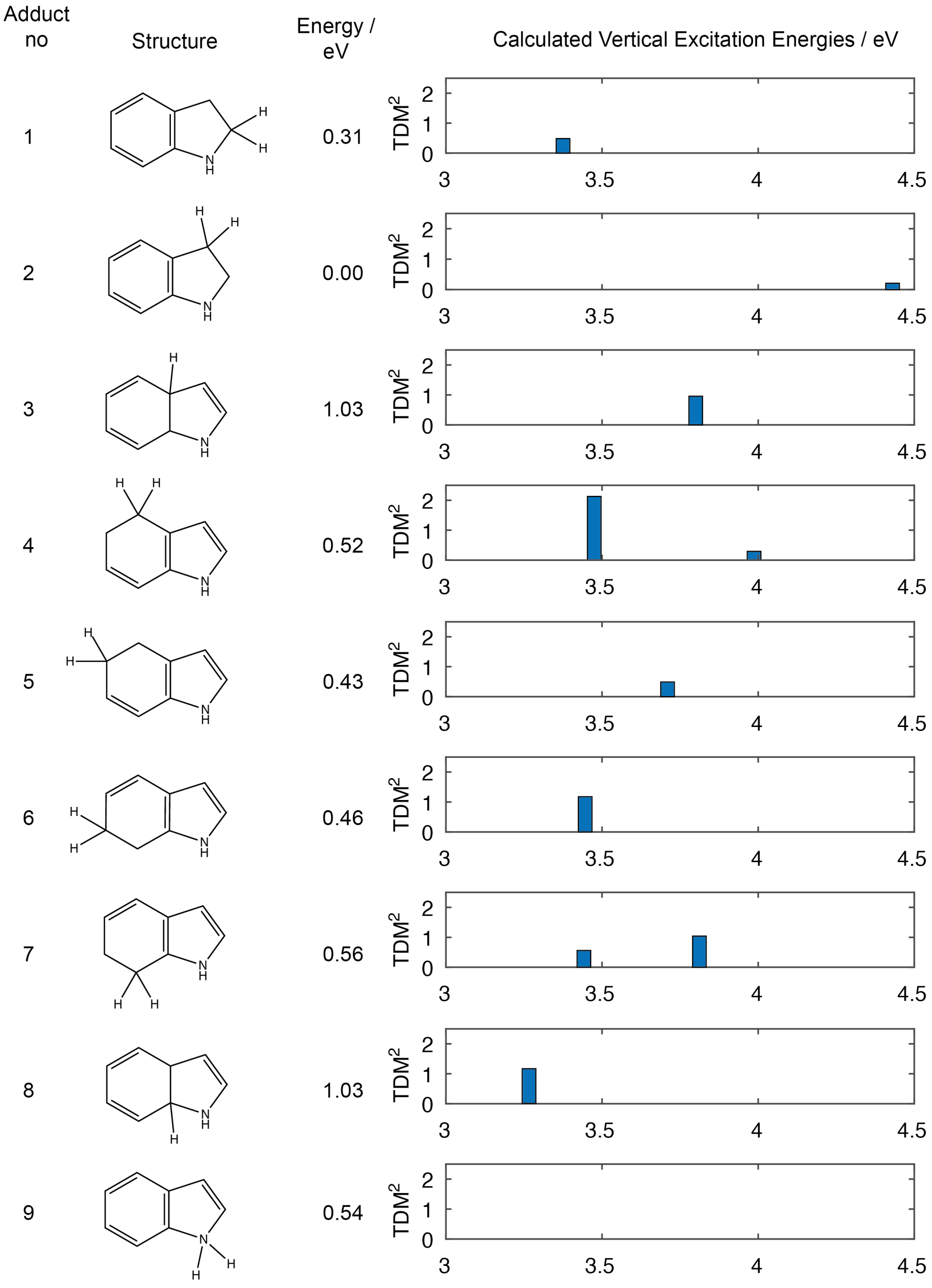

Figure S18. All possible adduct Indole cation–H atom isomers and associated energies (relative to adduct 2) optimized with MP2/aug-cc-pVTZ. The right-hand panel gives the calculated excited electronic states and associated transition dipole moments (a.u.) at the EOM-CCSD/aug-cc-pVDZ level of theory. It is important to note that all calculations were performed *in vacuo*, and thus for experiments in water, these energies are expected to red-shift. Further, several different adduct isomers may be formed depending on the direction the H atom attacks the indole cation.

8. 266 nm Nanosecond Transient Absorption Data

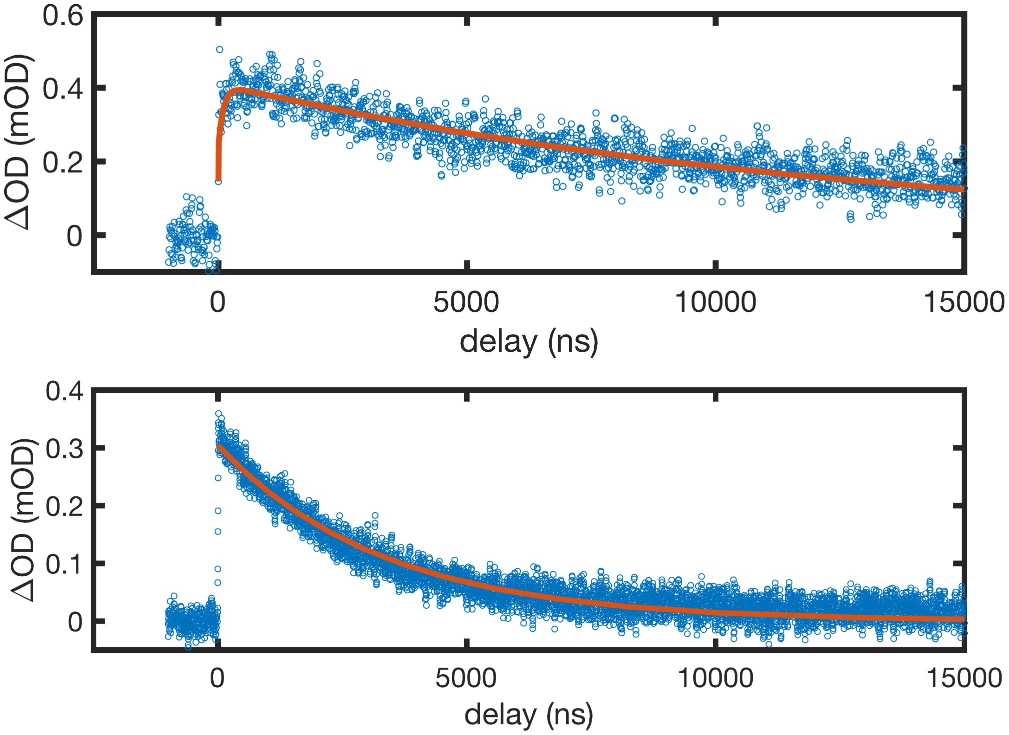

Figure S19: Excitation of 160 μM of de-aerated aqueous indole in a 1 cm long (probe path) flow cell illuminated with a 5 ns 266 nm pulse (0.5 cm pump path). With a broadband xenon lamp probe, transient absorption is recorded with an instrument response of ~ 5 ns. (Top) Photodiode response for a detected wavelength of 450 nm. Fitting is to a two-exponential rise (60% IRF limited and 40% ~ 100 ns) and a 13 μs single exponential decay (red line). (Bottom) Photodiode response for a detected wavelength of 700 nm. Fit (red line) is to a 3.3 μs single exponential decay. Both signals are strongly sensitive to dissolved oxygen.

9. 292 nm Aqueous Indole Transient Absorption

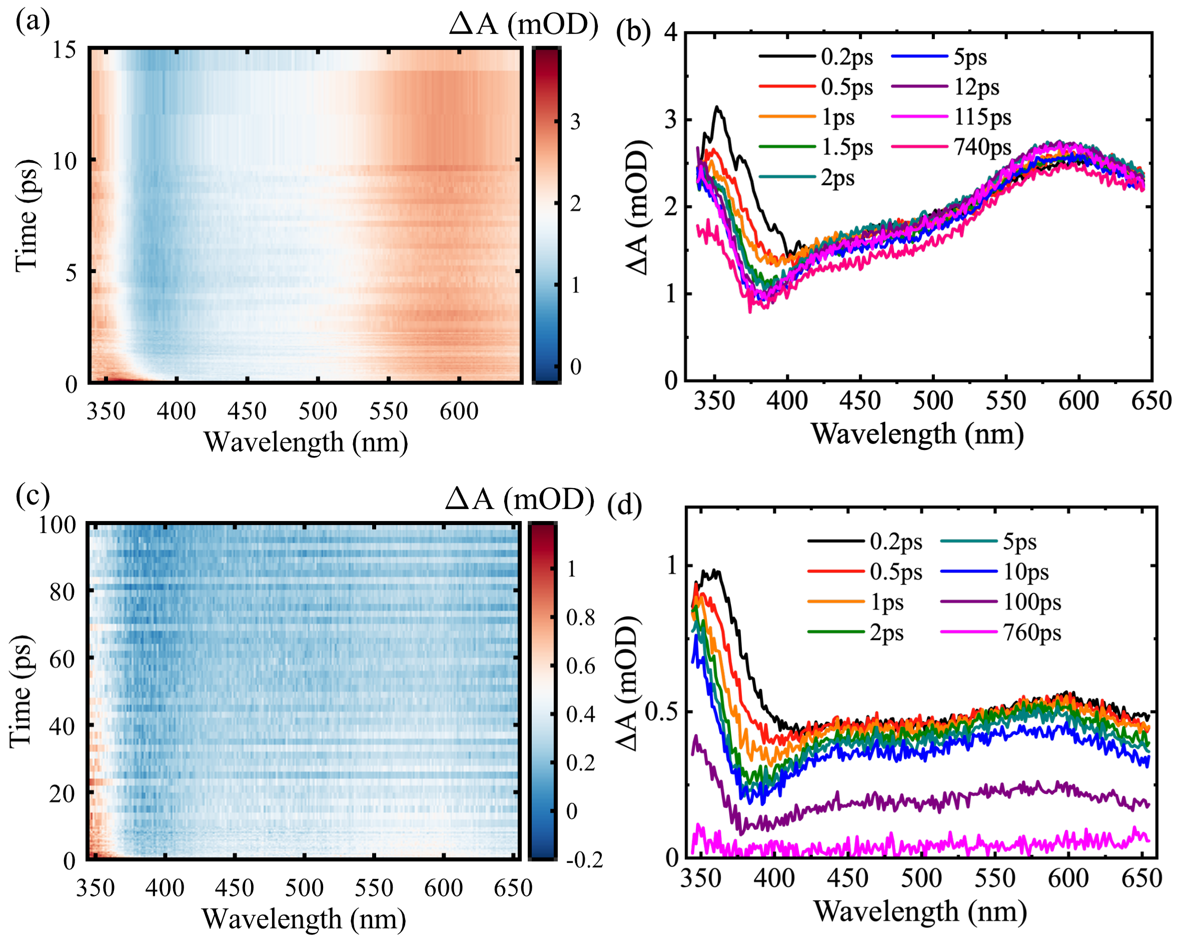

Figure S20: (a) Contour plot of the full 2D transient absorption data set of 17 mM indole in water at 292 nm (b) Spectral slices at a series of time delays (c) Contour plot of the TA data set when 0.5 M KNO_3 was added to the 10 mM aqueous indole (d) Spectral slices for the aqueous indole when 0.5 M KNO_3 is added to the solution.

The data in Fig. S20 should be compared with 268 nm excitation of aqueous indole in Fig. 4 of the main paper. The magnitude of spectral shifting of the S_1 ESA at ~370 nm in 292 nm pumped data is less pronounced (5–10 nm) than for 268 nm excitation (compare Fig. S20(b) with Fig. 4(b)), consistent with less internal vibrational energy cooling.

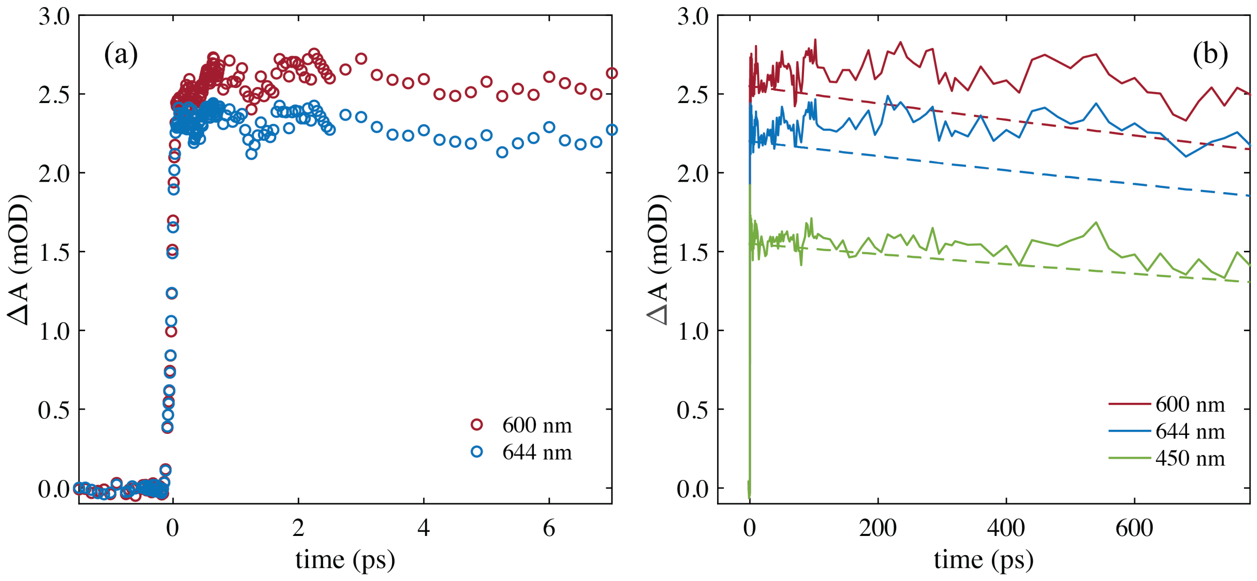

Figure S21. Comparison of kinetics for 292 nm indole in water for (a) early time delays and (b) over entire probe delay window.

Kinetics for 292 nm pumped TA data displayed in Fig. S21 appear very similar to those obtained with 268 nm irradiation (see Fig. S14). This indicates that the same photochemical dynamics apply at the two pump excitation wavelengths.

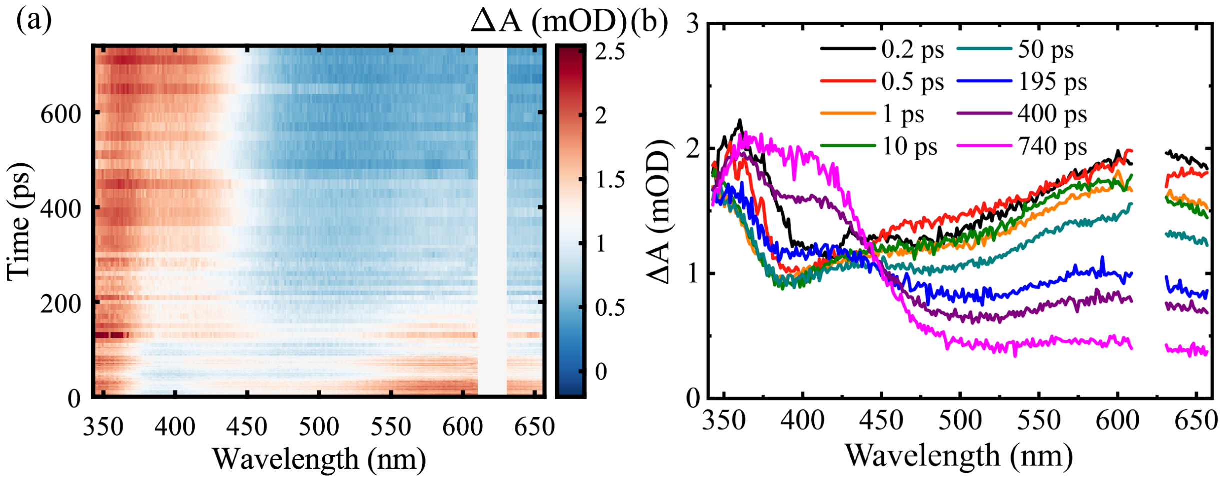

Figure S22. (a) Contour plot of the full 2D transient absorption data set of 17 mM indole in water at 292 nm with 0.2 M HCl (b) Spectral slices at a series of time delays. The signal in the region between 610 and 630 nm in panels (c) and (d) was removed due to the harmonic of pump scatter.

As for 268 nm, we carried out a second quenching experiment with HCl where H^+ can quench electrons either inside or outside the contact pair. Adduct formation is seen at the excitation wavelength also as well as loss of ESA.

To check the effect of H^+ on the fluorescence yield as a function of excitation energy, we recorded a fluorescence excitation spectrum in the presence and absence of protons. The quenching efficiency is invariant to the excitation wavelength between 260–290 nm.

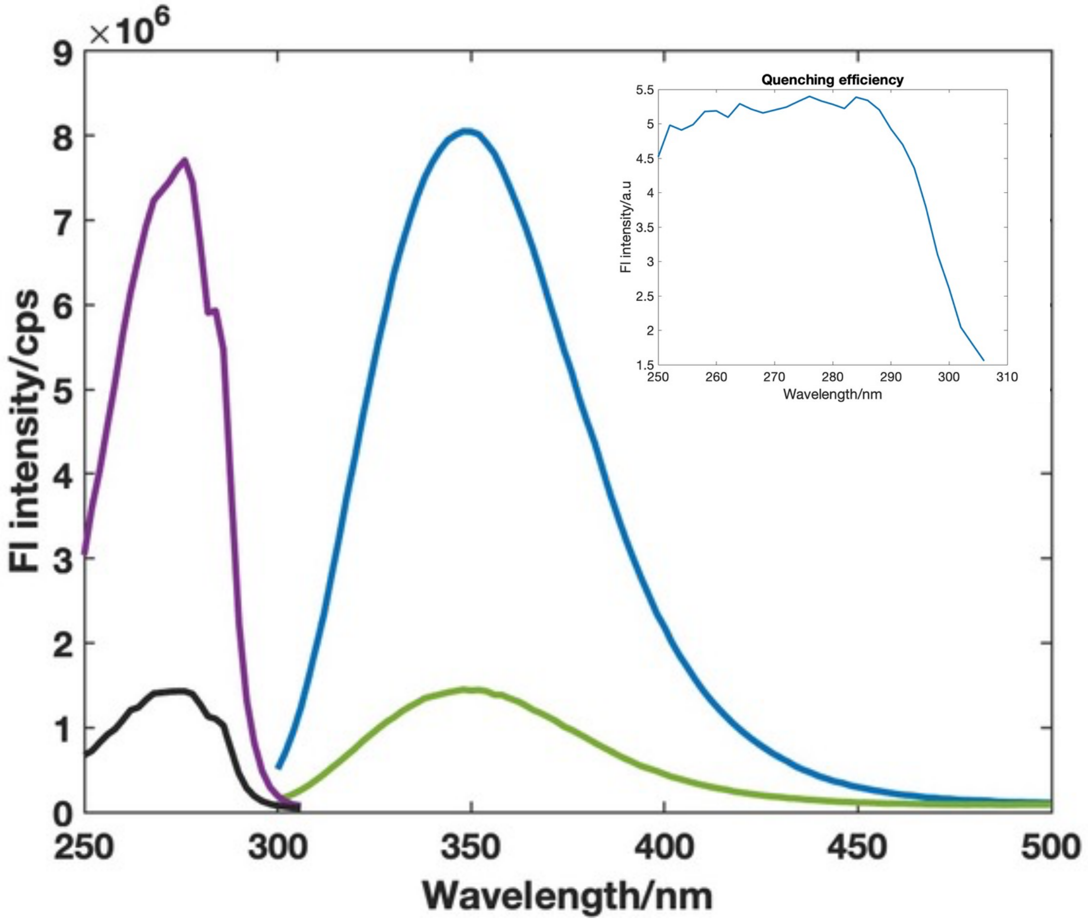

Figure S23. Fluorescence emission and excitation spectra acquired for 17 μM indole in water with 0.2 HCl or 0.2 KCl. Emission spectra were collected using 292 nm excitation. Excitation spectra were acquired for fluorescence at 350 nm. Excitation and emission bandwidths are 2 nm.

10. 255 nm Transient Absorption of Indole in Methanol

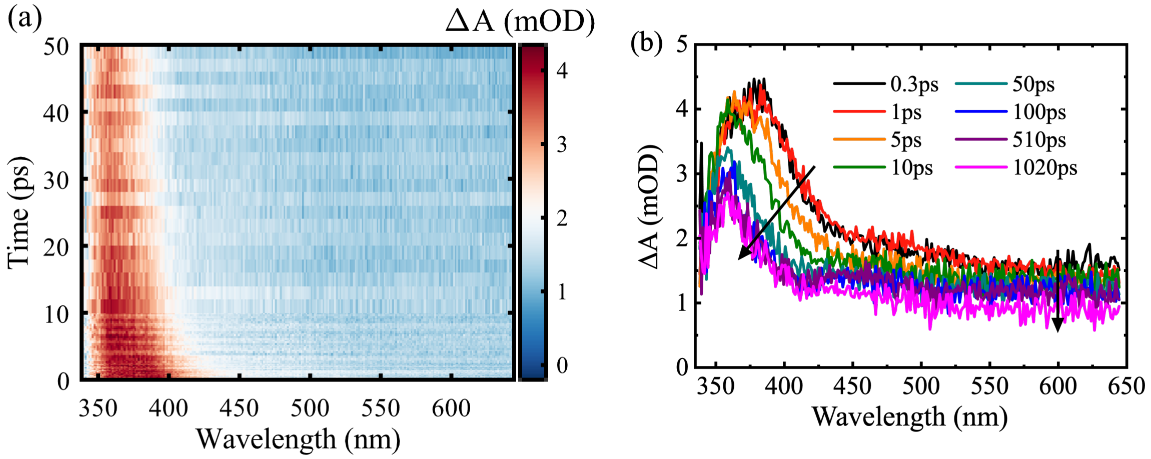

Figure S24. (a) False color contour plot of the full transient absorption spectrum of 20 mM indole in methanol using 255 nm excitation. (b) Spectral slices at a series of different time delays.

TA data acquired in methanol with 255 nm excitation (Fig. S24) show a strong resemblance to those acquired in ethanol recorded at 266 nm: the blue region of the TA data is dominated by a blue shift in the parent ESA at ~370 nm due to vibrational cooling on the S_1 potential. Notably absent from the data are spectral signatures of the indole cation (expected at ~575 nm) and solvated electron (~690 nm) indicating that photoionization does not occur in methanol solution.

References

1. Riedle, E.; Beutter, M.; Lochbrunner, S.; Piel, J.; Schenkl, S.; Spörlein, S.; Zinth, W. Generation of 10 to 50 fs pulses tunable through all of the visible and the NIR. *Appl. Phys. B* **2000,** *71*, 457-465.

2. Tauber, M. J.; Mathies, R. A.; Chen, X.; Bradforth, S. E. Flowing liquid sample jet for resonance Raman and ultrafast optical spectroscopy. *Rev. Sci. Instrum.* **2003,** *74*, 4958-4960.

3. Werner, H. J.; Knowles, P. J.; Knizia, G.; Manby, F. R.; Schütz, M. Molpro: a general-purpose quantum chemistry program package. *Wiley Interdiscip. Rev. Comput. Mol. Sci.* **2012,** *2*, 242-253.

4. Werner, H.; Knowles, P.; Knizia, G.; Manby, F.; Schütz, M.; Celani, P.; Györffy, W.; Kats, D.; Korona, T.; Lindh, R. MOLPRO, version 2015.1, a package of *ab initio* programs. *University of Cardiff, Cardiff, Wales, UK* **2015**.

5. Küpper, J.; Pratt, D.; Meerts, W. L.; Brand, C.; Tatchen, J.; Schmitt, M. Vibronic coupling in indole: II. Experimental investigation of the ^1L_a–^1L_b interaction using rotationally resolved electronic spectroscopy. *Phys. Chem. Chem. Phys.* **2010,** *12*, 4980-4988.

6. Brand, C.; Küpper, J.; Pratt, D. W.; Meerts, W. L.; Krügler, D.; Tatchen, J.; Schmitt, M. Vibronic coupling in indole: I. Theoretical description of the ^1L_a–^1L_b interaction and the electronic spectrum. *Phys. Chem. Chem. Phys.* **2010,** *12*, 4968-4979.

7. Kubota, M.; Kobayashi, T. Electronic structures of melatonin and related compounds studied by photoelectron spectroscopy. *J. Electron. Spectrosc. Relat. Phenom.* **2003,** *128*, 165-178.

8. Domelsmith, L.; Munchausen, L. L.; Houk, K. Photoelectron spectra of psychotropic drugs. 1. Phenethylamines, tryptamines, and LSD. *J. Am. Chem. Soc.* **1977,** *99*, 4311-4321.

9. Serrano-Andrés, L.; Roos, B. O. Theoretical Study of the Absorption and Emission Spectra of Indole in the Gas Phase and in a Solvent. *J. Am. Chem. Soc.* **1996,** *118*, 185-195.

10. Bräm, O.; Oskouei, A. A.; Tortschanoff, A.; van Mourik, F.; Madrid, M.; Echave, J.; Cannizzo, A.; Chergui, M. Relaxation Dynamics of Tryptophan in Water: A UV Fluorescence Up-Conversion and Molecular Dynamics Study. *J. Phys. Chem. A* **2010,** *114*, 9034-9042.

11. Kumar, G.; Roy, A.; McMullen, R. S.; Kutagulla, S.; Bradforth, S. E. The influence of aqueous solvent on the electronic structure and non-adiabatic dynamics of indole explored by liquid-jet photoelectron spectroscopy. *Faraday Discuss.* **2018,** *212*, 359-381.

12. He, L.; Tomaník, L.; Malerz, S.; Trinter, F.; Trippel, S.; Belina, M.; Slavíček, P.; Winter, B.; Küpper, J. Specific versus Nonspecific Solvent Interactions of a Biomolecule in Water. *J. Phys. Chem. Lett.* **2023,** *14*, 10499-10508.

13. Roy, A.; Seidel, R.; Kumar, G.; Bradforth, S. E. Exploring Redox Properties of Aromatic Amino Acids in Water: Contrasting Single Photon vs Resonant Multiphoton Ionization in Aqueous Solutions. *J. Phys. Chem. B* **2018,** *122*, 3723-3733.

14. Zhan, C.-G.; Dixon, D. A. The Nature and Absolute Hydration Free Energy of the Solvated Electron in Water. *J. Phys. Chem. B* **2003,** *107*, 4403-4417.

15. Gaiduk, A. P.; Pham, T. A.; Govoni, M.; Paesani, F.; Galli, G. Electron affinity of liquid water. *Nat. Commun.* **2018,** *9*, 247.

16. Tine, A.; Aaron, J.-J. A spectroscopic study of substituent and solvent effects on the luminescence of indoles. Correlations of the fluorescence and phosphorescence parameters with the sigma Hammett constants. *Can. J. Spectrosc.* **1984,** *29*, 121-130.

17. Sobolewski, A. L.; Domcke, W. Photoinduced charge separation in indole–water clusters. *Chem. Phys. Lett.* **2000,** *329*, 130-137.

18. Jaiswal, V. K.; Kabaciński, P.; Nogueira de Faria, B. E.; Gentile, M.; de Paula, A. M.; Borrego-Varillas, R.; Nenov, A.; Conti, I.; Cerullo, G.; Garavelli, M. Environment-Driven Coherent Population Transfer Governs the Ultrafast Photophysics of Tryptophan. *J. Am. Chem. Soc.* **2022,** *144*, 12884-12892.

19. Gryczynski, I.; Wiczk, W.; Johnson, M. L.; Lakowicz, J. R. Lifetime distributions and anisotropy decays of indole fluorescence in cyclohexane/ethanol mixtures by frequency-domain fluorometry. *Biophys. Chem.* **1988,** *32*, 173-185.

20. Chen, Y.; Liu, B.; Yu, H.-T.; Barkley, M. D. The peptide bond quenches indole fluorescence. *J. Am. Chem. Soc.* **1996,** *118*, 9271-9278.

21. Horne, G. P.; Pimblott, S. M.; LaVerne, J. A. Inhibition of radiolytic molecular hydrogen formation by quenching of excited state water. *J. Phys. Chem. B* **2017,** *121*, 5385-5390.
